# Thinned and Welded Silver Nanowires for Intelligent Pressure and Humidity Sensing Enabled by Machine Learning

**DOI:** 10.1002/advs.202507610

**Published:** 2025-08-27

**Authors:** Jiajun Fan, Tao Wan, Tao Yin, Ziheng Feng, Peiyuan Guan, Tianxu Huang, Chao Liu, Mengyao Li, Shuying Wu, Shuhua Peng, Shihao Huang, Zhaojun Han, Dongchen Qi, Wenlong Cheng, Dewei Chu

**Affiliations:** ^1^ School of Materials Science and Engineering The University of New South Wales Sydney NSW 2052 Australia; ^2^ School of Aerospace Mechanical and Mechatronic Engineering The University of Sydney Sydney New South Wales 2006 Australia; ^3^ School of Mechanical Engineering The University of New South Wales Sydney NSW 2052 Australia; ^4^ Eastern Institute of Technology Ningbo Zhejiang 315200 China; ^5^ School of Mechanical Medical and Process Engineering Queensland University of Technology Brisbane QLD 4000 Australia; ^6^ School of Chemical Engineering The University of New South Wales Sydney NSW 2052 Australia; ^7^ School of Chemistry and Physics Queensland University of Technology Brisbane QLD 4000 Australia; ^8^ Centre for Materials Science Queensland University of Technology Brisbane QLD 4000 Australia; ^9^ School of Biomedical Engineering The University of Sydney Darlington New South Wales 2008 Australia

**Keywords:** conductive network, machine learning, morse code, selective etching, sensing

## Abstract

The rapid development of flexible and wearable technologies urgently requires high‐performance and compatible flexible electrodes. Silver nanowires (AgNWs) are considered a promising conductive material, but their inherent structural drawbacks significantly hinder their widespread application. Here, an innovative and facile strategy is employed to simultaneously enhance the electrical and optical properties of AgNWs by precisely tuning their microstructure. Thinner AgNWs are achieved through the selective etching, alongside enhanced heating and mechanical properties, stemming from the welded structure and preserved conductive network integrity. A pressure sensor constructed with modified AgNWs demonstrates improved sensitivity compared to one using pristine AgNWs. By leveraging machine learning, the sensor can identify pressing behaviors with different fingers, achieving a high accuracy of 94.5%. The letters of the alphabet are also accurately recognized through analysis of unique resistance patterns in their Morse code. With the incorporation of a moisture‐sensitive graphene oxide layer, the device is capable of recognizing human respiration behaviors and detecting voices based on their distinctive respiration and pronunciation patterns. The strategy employed to tailor the functionality of AgNWs, combined with further integration of machine learning, presents a promising avenue for advancing flexible and wearable electronics.

## Graphical abstract

Thinned and welded silver nanowires are prepared by selectively engineering their morphology and structure. The resulting films show enhanced optical, electrical, thermal, and mechanical properties. By incorporating machine learning, the developed pressure and humidity sensors are able to accurately identify finger pressing behaviors, monitor human respiration, and detect voices.

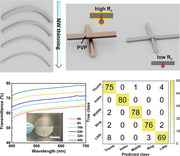

## 1 Introduction

Flexible and wearable devices that combine typical functional features (e.g., sensing and displaying) with excellent flexibility can be integrated into advanced platforms for applications in the Internet of Things (IoT) and healthcare monitoring.^[^ ^1^, ^2^, ^3^, ^4^ ^]^ The rapid advancement of artificial intelligence and machine learning technologies enhances the capability and efficiency of these systems, potentially revolutionizing our lifestyle by enabling personalized health monitoring and smart human movement detection.^[^ ^5^, ^6^, ^7^, ^8^ ^]^ Conductive electrodes are an essential component in numerous electronics and sensors, with transparent electrodes attracting significant interest for their distinct functionality and aesthetic prospects. Indium tin oxide (ITO) films are widely adopted as transparent conducting electrodes owing to their excellent optoelectrical characteristics. However, they face limitations for next‐generation flexible applications, including intrinsic rigidity that restricts mechanical deformation and high manufacturing costs associated with indium scarcity.^[^ ^9^, ^10^, ^11^ ^]^ Silver nanowires (AgNWs) electrodes feature uniquely interlocked networks that integrate high‐conductive NWs as building blocks with porous architectures designed to facilitate light transmission. Another notable advantage of AgNWs is their versatility, as they can function in rigid, flexible, stretchable, or even dynamic states when incorporated with specific substrates, enabling seamless integration into various systems for diverse application scenarios.^[^ ^12^ ^–^ ^18^ ^]^

The stable operation of flexible transparent electrodes for signal detection and visualization necessitates low resistance and reliable electrical and mechanical stability. It is noted that the NW junctions in conductive networks play a significant role in their overall performance as pristine NWs are often loosely contacted due to the formed polymer layer on NWs surfaces, resulting in poorly conductive paths.^[^ ^19^, ^20^, ^21^ ^]^ To construct an efficient and robust conductive AgNWs network, various strategies have been employed to tailor the film structure and composition. For instance, highly efficient networks are designed via controlling AgNWs orientation through leveraging centrifugal inertia force,^[^ ^22^ ^]^ direct writing,^[^ ^23^ ^]^ and interface assembly.^[^ ^24^ ^]^ Although the ineffective junctions of AgNW networks are greatly reduced, additional treatments are still required to further engineer the contact of AgNWs. Introducing a coating layer (e.g., metals, metal oxides, and 2D materials) on AgNWs surfaces or junctions has shown great potential for improving wire‐to‐wire connections and enhancing their electrical and mechanical properties.^[^ ^25^, ^26^, ^27^, ^28^, ^29^, ^30^, ^31^, ^32^ ^]^ However, complex processes, such as sputtering and electrodeposition, along with precise surface engineering (e.g., coating thickness and interaction), are generally involved to prepare desirable AgNWs composite electrodes suitable for specific applications. The selection of coating materials also requires careful consideration to avoid sacrificing other attributes like optical transparency. Other current methods aimed at selectively welding AgNWs junctions involve the use of external stimuli, such as light‐induced plasmon resonance,^[^ ^33^, ^34^, ^35^ ^]^ mechanical pressing,^[^ ^36^, ^37^ ^]^ thermal annealing,^[^ ^38^, ^39^ ^]^ and chemical exposure.^[^ ^40^, ^41^, ^42^ ^]^ Yet these approaches rely on specialized equipment and face challenges in advancing the overall properties of AgNWs for high‐performance and intelligent flexible devices.

Herein, we proposed a new strategy to concurrently etch the surface and weld the junctions of AgNWs based on a facile solution treatment. Higher optical transparency and electrical conductivity are demonstrated in the designed AgNWs networks due to thinner NW structures and more efficient conductive pathways. With the aid of machine learning, the constructed pressure sensor using modified AgNWs can recognize different pressing behaviors and the letters of the alphabet with excellent accuracy. By combining the electrode with a moisture‐sensitive layer, the resulting humidity sensor demonstrates distinct performance under different human respiration patterns. Moreover, it successfully distinguishes voices through the evaluation of pronunciation patterns via machine learning. This work not only provides an effective way to engineer the device structure but also offers insights into the synergetic integration of sensors with machine learning for intelligent applications.

## 2 Results and Discussion

**Figure 1:**
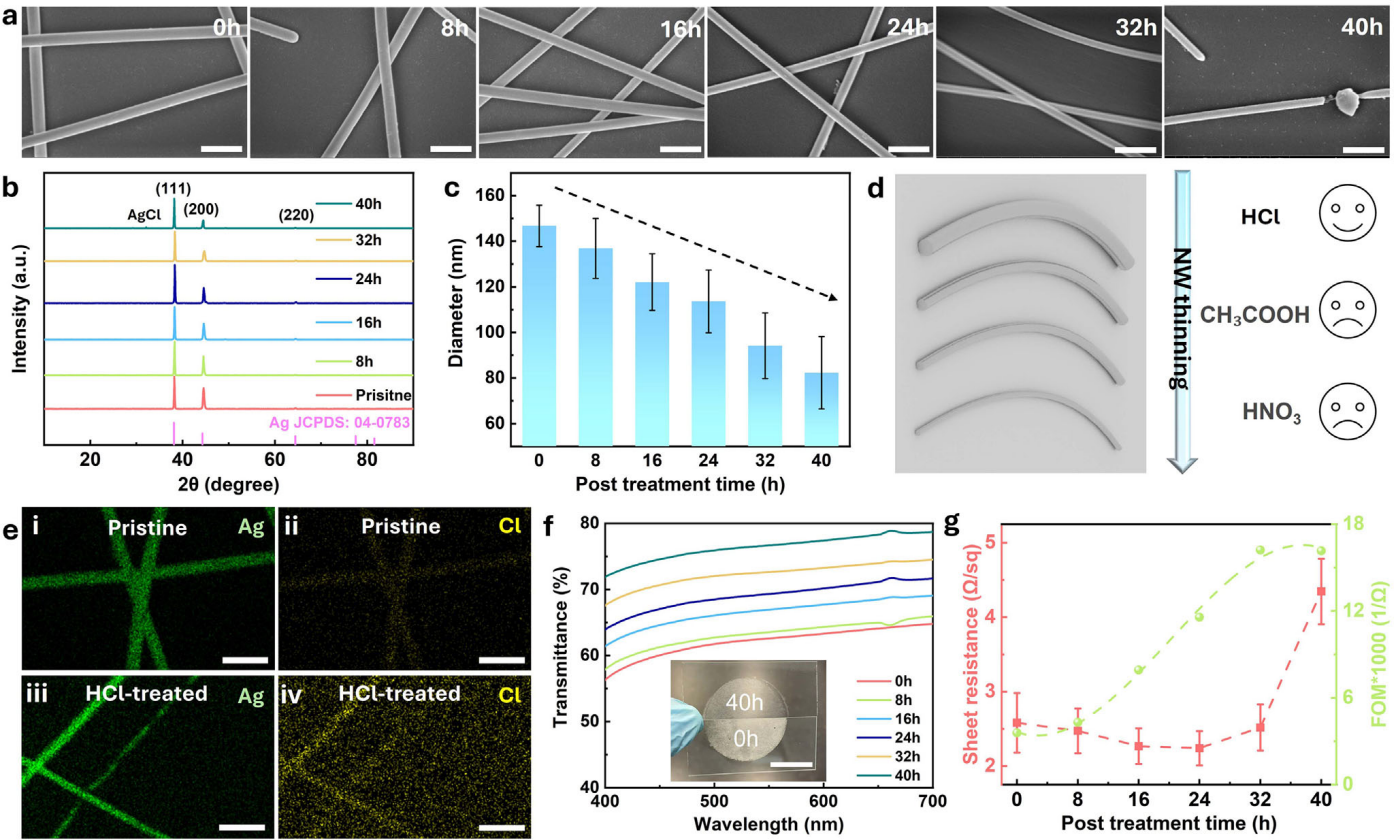
a) SEM images of pristine and HCl‐treated AgNWs. Scale bar: 500 nm. b) XRD patterns of pristine and HCl‐treated AgNWs. c) Average diameters of pristine and HCl‐treated AgNWs. d) Schematic of AgNWs after acid treatments. e) EDS element mapping of i, ii) pristine and iii, iv) HCl‐treated AgNWs. Scale bar: 500 nm. f) Transmittance of films based on pristine and HCl‐treated AgNWs (excluding the substrate). The inset photograph shows AgNWs electrodes treated with and without HCl. Scale bar: 2 cm. g) The corresponding sheet resistance and FOM of AgNWs films.

To investigate the morphological evolution of AgNWs under acidic conditions, the effects of HCl treatment as well as comparative treatments using acetic acid and nitric acid were systematically examined. Previous studies have demonstrated that sodium halide salt solution,^[^ ^41^ ^]^ H_2_O_2_ vapor,^[^ ^43^ ^]^ HCl vapor,^[^ ^44^ ^]^ HCl solution,^[^ ^45^ ^]^ and acidic sol‐gel treatment^[^ ^46^ ^]^ can effectively induce AgNW welding, thereby enhancing electrical conductivity. This welding process is generally attributed to the dissolution of Ag into Ag⁺ at the nanowire surface upon chemical exposure, followed by localized redeposition of Ag at high‐curvature junctions. The resulting metallic bridges reduce inter‐nanowire contact resistance and stabilize the network. However, unlike previous reports that primarily focused on short‐term welding effects, our study reveals that prolonged HCl exposure induces a gradual and controllable morphological transformation, in which junction welding is accompanied by uniform thinning of the AgNWs. This dual process leads to a simultaneous enhancement of optical transmittance and electrical performance, a synergy that has rarely been discussed in previous studies. Scanning electron microscopy (SEM) was used to elucidate the etching process of AgNWs at room temperature by HCl solution immersion. With an increase in the treatment duration from 8 to 40 h, a reduction in the diameter of AgNWs is clearly observed (**Figure** **1**a). In addition to the observed thinning effect, transmission electron microscopy (TEM) analysis reveals that the surface morphology of AgNWs undergoes noticeable changes after extended HCl treatment. As shown in Figure S1 (Supporting Information), the 40h‐treated AgNWs exhibit a roughened and uneven texture, indicating excessive etching. Such etching influences the contact between AgNWs, posing a risk of structural instability. These findings highlight the importance of optimizing the HCl treatment duration, as excessively long exposure time may compromise the integrity of AgNWs. As a result, an appropriate treatment duration is required, providing an optimal balance between thinning, welding, and maintaining network stability. X‐ray diffraction (XRD) was used to study the crystal structures of AgNWs (Figure 1b). All samples exhibit two characteristic peaks at ≈38.1° and 44.3°, attributed to the (111) and (200) planes of cubic phase Ag (JCPDS 04‐0783), respectively. The decrease in the relative intensity of the peak at 44.3° indicates that HCl preferentially etches the surface of AgNWs, and a much lower intensity is observed in the sample after 40 h treatment due to a longer reaction time, which is consistent with the previous study.^[^ ^44^ ^]^ A minor peak of AgCl at 32.2° is also observed in AgNWs‐40 h. Figure 1c and Figures S1–S3 (Supporting Information) showed the microstructure and distribution of diameters and lengths of AgNWs after HCl treatments. An insignificant change of NW length is observed after HCl treatments, whereas the NW diameter significantly decreases from ≈147 to ≈ 82 nm. As a comparison, AgNWs were also treated with acetic acid and nitric acid, but no similar phenomenon was observed (Figure 1d; Figures S4 and S5, Supporting Information). In addition, energy‐dispersive X‐ray spectroscopy (EDS) elemental mapping further confirms the strong Cl signal in the treated AgNWs (Figure 1e; Figure S6, Supporting Information). The results clearly suggest that Cl^−^ ions have a significant effect on the etching process (will discuss later).

The optical properties of AgNW‐based films were also studied, as shown in Figure 1f. The films were fabricated using a vacuum filtration method and subsequently subjected to HCl treatment with varying exposure durations, ranging from 0 to 40 h in 8‐h increments, to investigate the effects on optical transparency. Notably, the small anomaly near 660 nm in the transmittance spectra is attributed to a lamp‐switching artifact commonly observed in dual‐source UV–vis spectrophotometers, and does not reflect any intrinsic optical response of the AgNW films. The transmittance spectra indicate a progressive increase in optical transparency with prolonged HCl treatment, with the transmittance at 550 nm rising from 62.6% (0 h) to 76.7% (40 h). This enhancement is attributed to the controlled thinning of AgNWs, which reduces light scattering while maintaining electrical connectivity. The inset image further illustrates this trend, showing an increasingly transparent film after extended acid exposure. These findings demonstrate the effectiveness of HCl treatment in optimizing AgNW networks for high‐performance transparent electrodes. Meanwhile, AgNW networks exhibit a correlation in sheet resistance with the duration of HCl treatment. As shown in Figure 1g, pristine AgNWs show a sheet resistance of 2.6 Ω sq^−1^, which gradually decreases with increasing treatment time. However, a longer treatment (e.g., 40 h) could cause over‐etching, resulting in partial NW disconnection (Figure 1a) and thereby a substantial rise in resistance. The figure of merit (FOM) is utilized to evaluate their overall performance and is calculated as FOM = *T^10^/R_s_*, where *T* is the optical transmittance at 550 nm and *R_s_* ​is the film sheet resistance.^[^ ^30^ ^]^ The modified AgNWs have a much higher FOM than pristine AgNWs, further confirming a better optoelectronic performance.

**Figure 2:**
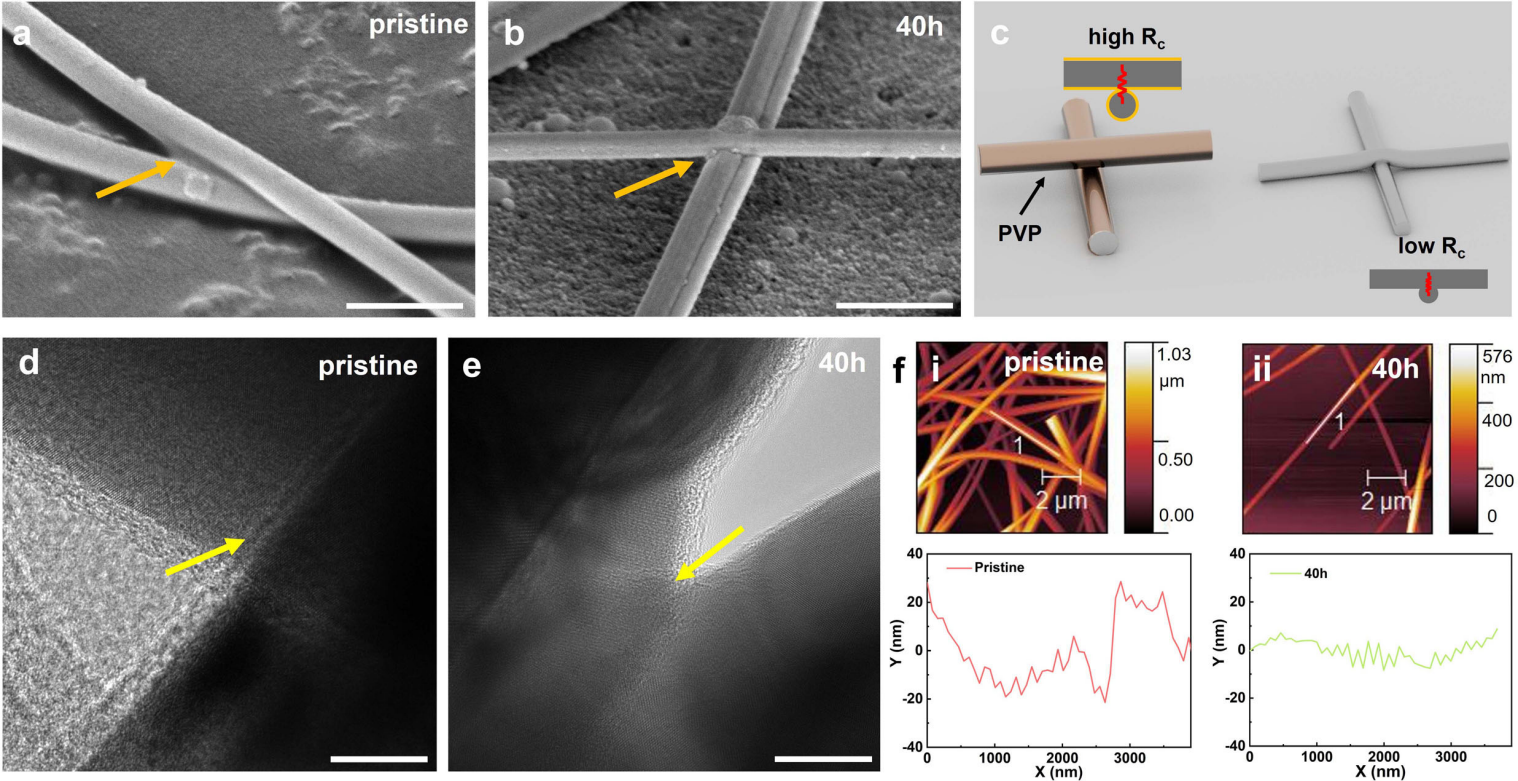
a,b) Tilted SEM images of pristine and treated AgNWs. Scale bar: 500 nm. c) Schematic of contacted AgNWs with low and high R_c_. d,e) TEM images of pristine and treated AgNWs. Scale bar: 10 nm. f) AFM images of i) pristine AgNWs and ii) AgNWs‐40 h. The roughness spectra correspond to the line scan results.

Tilted SEM images in **Figure** **2**a,b reveal that pristine AgNWs are wrapped with organic residue and loosely connected, while a close contact or even welding is observed in the treated NWs, giving rise to a low contact resistance (R_c_) (Figure 2c). This is further validated by the TEM images of AgNWs (Figure 2d,e), which align with SEM observations of the contacts at NW junctions. It should be noted that while the SEM and TEM images suggest enhanced NW contact after HCl treatment, they do not provide atomic‐scale confirmation of junction fusion. Therefore, the term “welding” in this work is used to describe improved morphological connectivity and electrical performance, rather than direct evidence of atomic‐level fusion. During HCl treatments, the residue PVP capping layer on AgNWs can be easily detached, caused by the reaction between Cl^−^ ions and Ag^+^ ions,^[^ ^30^, ^47^ ^]^ which could expose more active Ag surface for the etching. The exposed Ag reacts with oxygen from air in the HCl solution to form silver oxide (Ag_2_O) (Equation 1). When in contact with HCl solution, the Ag_2_O layer reacts to generate silver chloride (AgCl) (Equation 2), which can further be transformed back to Ag atoms (Equation 3) due to the light‐sensitive characteristic.^[^ ^44^, ^48^ ^]^

(1) $$4Ag+O_{2}\to 2Ag_{2}O$$

(2) $$Ag_{2}O+2HCl\to 2AgCl+H_{2}O$$

(3) $$2AgCl\to 2Ag+Cl_{2}$$

The generated Ag atoms then redeposit in a nearby area, particularly at the junctions between AgNWs with a heightened reactivity,^[^ ^40^, ^49^ ^]^ leading to the welding of AgNWs. The etching reaction is highly related to the immersion time, and thinner NWs are prepared after a longer treatment period. However, a low‐concentration HCl solution (0.1 m) could not achieve the desired reactivity (Figure S7, Supporting Information). On the other hand, Ag can be easily converted into AgCl or even detached from the substrate due to excessive etching using 32 wt.% HCl (Figure S8, Supporting Information). These results underscore the pivotal role of HCl treatment in influencing the network structure of AgNWs as well as the importance of meticulous treatment for effective nanowelding and functional integrity. The atomic force microscopy (AFM) results in Figure 2f indicate a decrease in the average surface roughness after HCl treatment.^[^ ^50^ ^]^ However, it should be noted that the line scan range (≈4 µm) was narrower than the full AFM image (≈10 µm), which may affect the representativeness of the extracted roughness values. The observed decrease in surface roughness is likely influenced by both nanowire thinning and reduced surface coverage after acid treatment.^[^ ^51^ ^]^ In addition to enhancing surface smoothness and optical transmittance, the acid treatment process used in this study is mild, low‐temperature, and solution‐based, making it well‐suited for integration with flexible substrates and scalable fabrication methods. Its compatibility with ambient processing conditions suggests potential for application in large‐area electronics using roll‐to‐roll or printing techniques. Future work will explore the adaptation of this treatment for continuous and high‐throughput manufacturing platforms.

**Figure 3:**
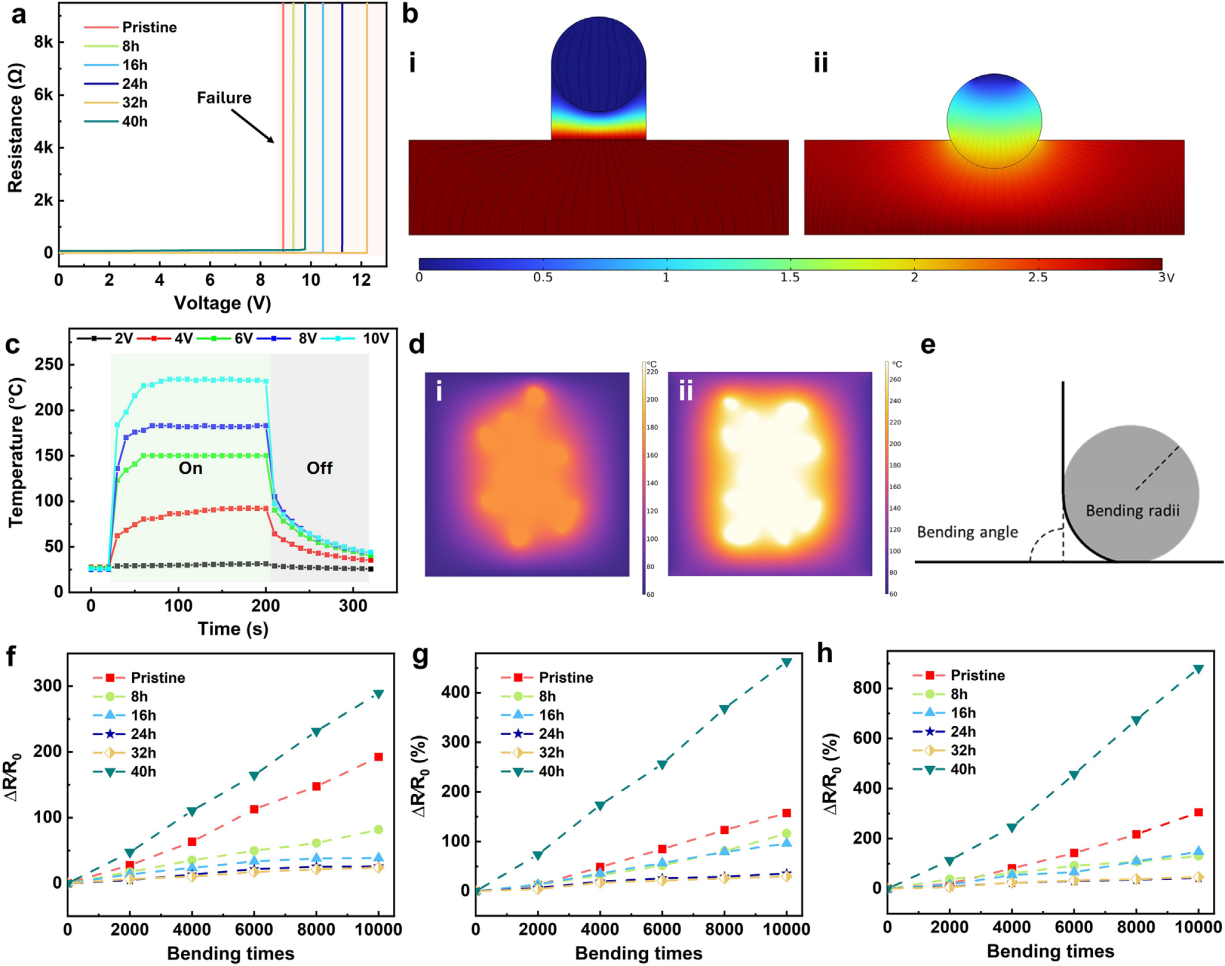
a) Resistance evolution of AgNWs with varying HCl treatments under an electric field. b) Simulated electric potential distribution of i) separated and ii) contacted AgNWs. c) Heating performance of acid‐treated AgNWs (32 h). d) Simulated temperature distribution of i) pristine AgNWs and ii) acid‐treated AgNWs before failure. e) Diagram detailing the bending test parameters. Relative resistance evolution of AgNWs subjected to 10 000 bending cycles at bending radii of f) 10 mm, g) 5 mm, and h) 2.5 mm.

To evaluate the reliability of AgNWs‐based electrodes for practical application, a voltage ramp test (0.5 V min^−1^) was carried out (**Figure** **3**a). The resistances of all samples show a sharp change during device failure. As the applied voltage increases, the Joule heating effect can induce a temperature rise, potentially leading to thermal degradation of AgNWs networks. This is particularly pronounced at the junctions where current density is typically higher, facilitating the formation of hot spots and thermal breakdown. Additionally, under substantial electric fields, electromigration may occur, where metal ions migrate, leading to material displacement and creating voids or hillocks that interrupt electrical pathways (Figure S9, Supporting Information).^[^ ^52^, ^53^ ^]^ The HCl treatment modifies the surface of AgNWs and promotes nanowelding at the junctions, which effectively reduces the contact resistance between AgNWs.^[^ ^54^ ^]^ As a result, enhanced voltage stability is achieved in HCl‐treated samples. Nevertheless, extensive etching (40 h) leads to significant junction damage, resulting in an earlier electrical failure under applied voltage. COMSOL was used to simulate the electric potential distribution of AgNWs with and without a gap (Figure 3b). In contrast to fully contacted AgNWs, the electric potential concentrates at the gap between AgNWs, which could induce localized melting and AgNWs damage.^[^ ^31^ ^]^ Owing to the better electrical stability, the modified AgNWs show enhanced heating performance (Figure 3c,d; Figure S10, Supporting Information). For example, AgNWs etched for 24 and 32 h realize a high temperature of >200 °C under 10 V, outperforming the pristine sample. The device was also used to heat water and ice (Figure S11, Supporting Information), demonstrating the potential applicability.

For practical applications, the mechanical durability of flexible electrodes is of paramount importance, which was systematically investigated using bending tests under varying conditions. These tests comprised 10 000 consecutive cycles with bending radii of 2.5, 5, and 10 mm, with the bending angle varying from 0° to 90° (Figure 3e). Figure 3f–h illustrates the sheet resistance changes in AgNWs subjected to different bending radii. The etched samples (e.g., 24 and 32 h) exhibit superior mechanical stability compared to pristine AgNWs, highlighting the electrodes’ ability to withstand repeated mechanical deformation. For the pristine sample, resistance increase under moderate bending may result from variable interfacial adhesion or local delamination during testing, rather than the bending radius itself. This improvement is consistent with previous studies on chemically welded AgNW networks, where enhanced junction connectivity contributed to greater mechanical durability under strain.^[^ ^55^ ^]^ The acid treatment in our work similarly reinforces the nanowire network, leading to improved strain tolerance without compromising flexibility.

However, the sample treated for 40 h exhibits a substantial increase in resistance (>200%), particularly under a small bending radius. This is ascribed to the fact that more acute bending places greater mechanical stress on the over‐etched AgNWs, exacerbating the deformation or disconnection of AgNWs and thereby disrupting the conductive pathways.

**Figure 4:**
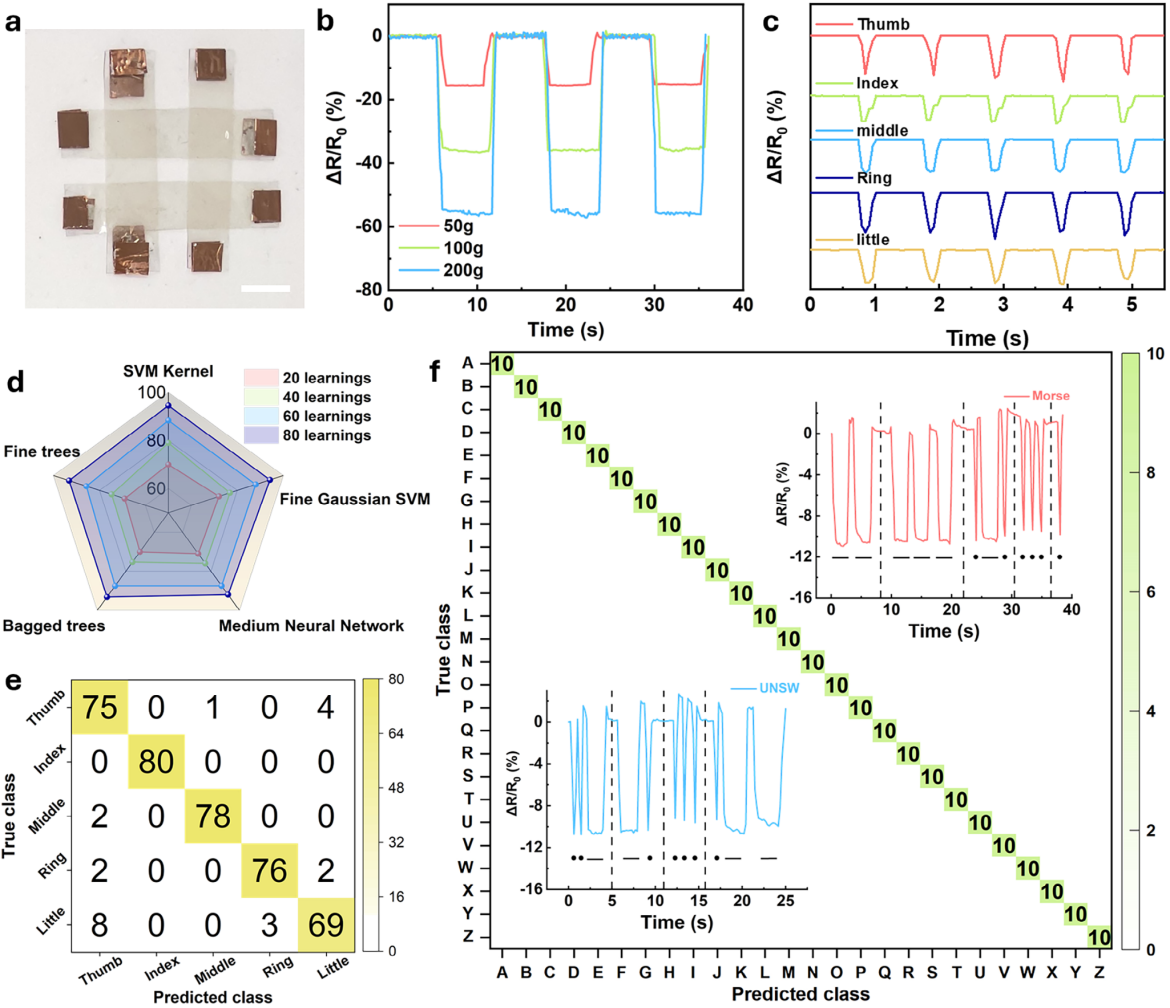
a) A photograph of AgNWs sensors. Scale bar: 1 cm. b) Relative resistance changes induced by different weights applied to the sensor fabricated from AgNWs treated with HCl for 32 h. c) The relative resistance changes of the pressure sensor with five different fingers. d) Radar plot representing the total accuracy of five fingers with various machine learning models. e) Confusion matrix after 80 training iterations. f) Alphabet classification showing the accuracy of distinguishing each letter after 10 sets of training iterations. The insets show the resistance changes representing the Morse codes of “UNSW” and “Morse”.

In addition, AgNWs‐based pressure sensors were also fabricated (**Figure** **4**a), and their overall resistances are highly related to the contact between the two AgNWs films (Figure S12, Supporting Information). Accordingly, higher conductivity is expected in the device with a closer NW contact. Figure 4b and Figure S13 (Supporting Information) illustrate the pressure response of sensors based on HCl‐treated and pristine AgNWs, respectively. The HCl‐treated sensor exhibits a significantly higher relative resistance change (ΔR/R_0_) under the same loading conditions compared to the pristine counterpart. This enhanced performance is attributed to the improved junction connectivity and reduced contact resistance induced by the HCl‐driven thinning and welding process.^[^ ^56^, ^57^ ^]^ These results confirm that even with a modest reduction in sheet resistance, the morphological transformation leads to a substantial improvement in pressure sensing capability. Response time measurements indicate minimal change, with untreated AgNWs at 1 s and treated samples at 0.94 s, suggesting that the HCl treatment does not significantly impact the response speed (Figure S14, Supporting information). Figure 4c presents the resistance variations for different fingers pressing on the sensor, correlating with the pressing behaviors illustrated in Figure S15 (Supporting Information). The resistance‐time profiles under finger pressing exhibit a consistent waveform and peak magnitude over several cycles. In addition, the rising and falling edges of the signal show good symmetry between the loading and unloading phases. However, the recorded resistance change (ΔR/R_0_) is influenced by the contact area, pressing angle, and force distribution of each finger. The thumb exhibits narrower peaks due to its side‐contact pressing posture rather than full fingertip contact. This results in a smaller effective contact area and more focused pressure application, leading to sharper resistance transitions. The index and middle fingers generate broader and more uniform peaks, as they apply force with a well‐distributed fingertip contact, leading to more stable resistance changes. The ring finger produces the highest resistance change, which is attributed to partial palm elevation during pressing. This alteration in hand posture redistributes the applied force, momentarily increasing the localized pressure on the sensor. The little finger shows the most irregular resistance pattern, likely due to its weaker pressing force and unstable contact, making its signal less consistent compared to other fingers. These variations in resistance response confirm that each finger generates a distinct pressure signature, which can be effectively distinguished by the sensor. This differentiation capability is particularly useful for applications in biometric authentication, personalized gesture recognition, and intelligent touch‐sensitive interfaces.

With the assistance of machine learning, the pressure sensor could identify which finger is pressing on it. Various machine learning methods, including Support Vector Machine (SVM) kernel, Fine Gaussian SVM, Bagged Trees, Fine Trees, and Medium Neural Network, were employed to evaluate the performance.^[^ ^58^, ^59^ ^]^ Figure 4d shows a radar chart comparing these machine learning models with the highest accuracy rates across 20, 40, 60, and 80 learning iterations. At 20 iterations, the accuracy rates of the models are ≈70%. By increasing the iterations from 20 to 60, the accuracy rates are significantly increased, reaching a range between 85% and 89%. Finally, at 80 iterations, the accuracy rates of SVM kernel, Fine Gaussian SVM, Bagged Trees, Fine Trees, and Medium Neural Network are 94.5%, 94.2%, 92%, 93.2%, and 93.2%, respectively. This highlights the progressive improvement in model performance with more training iterations, with the SVM kernel showing the highest accuracy at 80 iterations. SVM is a powerful machine learning algorithm used for classification tasks. It constructs an N‐dimensional hyperplane that optimally separates the data into different classes. It achieves high classification accuracy, particularly with small to medium‐sized datasets.^[^ ^60^ ^]^ In this experiment, MATLAB's Classification Learner Toolkit was used to develop machine learning models. This interactive tool simplifies the process of creating, training, and optimizing various predefined models. The AgNWs sensors demonstrated the ability to convert raw electrical signals into distinguishable data, enabling effective interpretation and response with machine learning.^[^ ^61^ ^]^

As shown in Table S1 (Supporting Information), our work achieves a higher classification accuracy (94.5%) with fewer training iterations (80 iterations) compared to similar studies that require more extensive training. This efficiency is attributed to our optimized AgNWs and advanced machine learning approach. While other studies, such as those using Random Forest or Artificial Neural Networks (ANN), achieve high accuracy, they often require large datasets (over 30 0000 samples) and significantly more training iterations. In contrast, our model utilizes a more efficient SVM Kernel, allowing for high accuracy with a reduced training dataset. It should be noted that differences in dataset size, model architecture, and training protocols among the listed studies may influence the reported accuracy values, and direct normalization is not feasible due to inconsistent reporting. Therefore, Table S1 (Supporting Information) serves as a qualitative comparison to illustrate representative strategies in machine learning‐assisted sensing.

Figure 4e and Figure S16 (Supporting Information) present confusion matrices generated by the SVM kernel across different training stages. The classification accuracy of the model can be greatly enhanced with increasing training iterations. To further aid understanding, a schematic illustration of the classification pipeline is provided in Figure S17 (Supporting Information), which visualizes how finger pressing signals are collected by the sensor and processed through the machine learning to yield class‐specific outputs. The classification accuracy of the model using five different fingers (thumb, index, middle, ring, and little) across these training iterations is also presented. At 80 iterations, the highest accuracy rates are recorded: 93.75% for thumb, 100% for index, 97.5% for middle, 95% for ring, and 86.25% for little, resulting in an overall accuracy of 94.5%. The result demonstrates the model's enhanced ability to accurately identify the finger being used for pressing with more training. Through the application of machine learning (SVM kernel model), the pressure sensor was also adopted to generate and interpret Morse code signals. Figure S18 (Supporting Information) displays the relative resistance changes corresponding to the Morse code for the 26 letters of the alphabet. The confusion matrices (Figure 4f) obtained after 10 sets of training iterations demonstrate the machine learning model's ability to correctly classify each letter based on the distinct resistance patterns produced by the pressure sensor (Figure S18, Supporting Information). In addition, the relative resistance changes for the Morse code representations of the words “UNSW” and “Morse” are also provided (Figure 4f).

**Figure 5:**
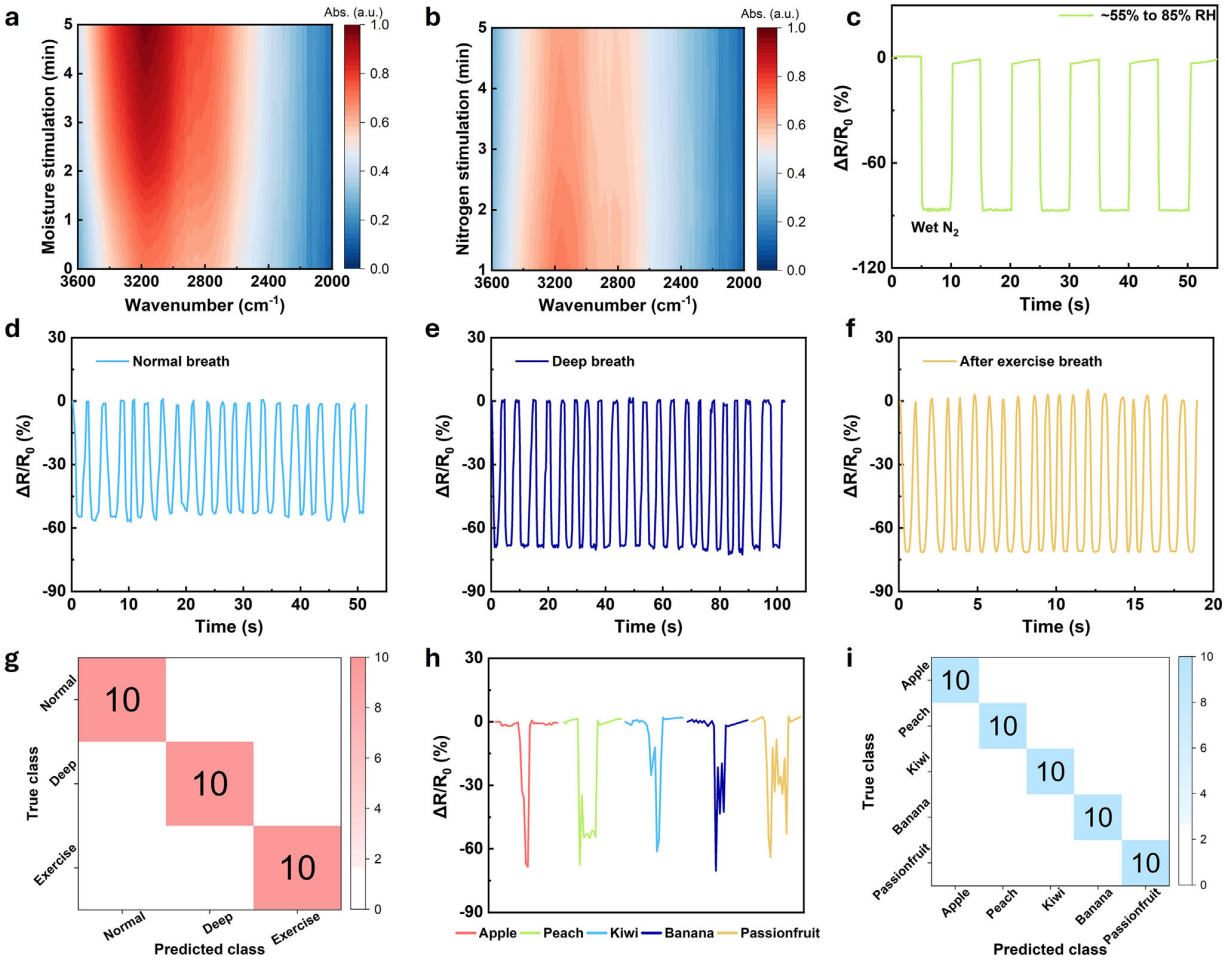
FTIR analysis of GO film under a) moisture exposure and b) dry N_2_ blowing over time. c) Relative resistance changes of the sensor under different humidities controlled by wet N_2_. Sensor response under d) normal breath, e) deep breath, and f) breath after exercise. g) Confusion matrix with 10 iterations of each breath mode. h) Resistance variation of the sensor triggered by different voices. i) Confusion matrix of voice classification.

Given the practical importance of humidity sensing in health and environmental monitoring, the designed AgNWs were integrated with hydrophilic GO as humidity sensors. The functional groups (e.g., carboxyl groups) in GO films will dissociate upon moisture exposure, releasing mobile charge carriers. A high film resistance is achieved at low relative humidity (RH) due to suppressed movement of the charge carriers, whereas low resistances are obtained at higher humidity levels caused by the facilitated ion migration.^[^ ^62^, ^63^ ^]^ The Fourier Transform Infrared (FTIR) spectra of GO film show the characteristic peak at ≈3150 cm^−1^, corresponding to the ‐OH absorption bond (**Figure** **5**a,b; Figure S19, Supporting Information). It is noted that the peak intensity gradually increases with prolonged moisture exposure time, and an opposite trend is observed upon moisture removal, which is consistent with previous studies.^[^ ^64^, ^65^ ^]^ The results clearly indicate that the sensing performance of GO films is highly associated with water‐related dynamics (i.e., adsorption/desorption of water molecules). The sensor's relative resistance change in response to different humidity levels was measured by blowing wet and dry N_2_ gas (Figure 5c; Figure S20, Supporting Information). Following dry gas blowing, the RH decreases from the ambient level of ≈55% to < 5%, resulting in a substantial increase in resistance. In addition, a relative resistance decrease of ≈87% is also achieved when the humidity is increased from ≈55% to ≈85% RH. Owing to the excellent moisture sensing performance, such a sensor can recognize human breathing behaviors in different conditions, as shown in Figure 5d–f. For normal breathing, the sensor's resistance decreases by ≈55% with an average duration of ≈2.6s per breath. During deep breathing, it shows a ≈68% decrease in resistance over 4.9s. After exercise, the resistance drops by ≈71% with a breath duration of 0.95s. These changes in resistance are due to the different absorption and desorption behaviors of water molecules in the GO film during exhalation and inhalation. It can be further utilized to accurately detect and differentiate distinct breathing intensities and durations for recognition of breathing patterns by leveraging machine learning (SVM kernel model, Figure 5g). The resistance patterns corresponding to these words are presented in Figure 5h, serving as the dataset used for classification in Figure 5i. Moreover, the sensor can be applied for human‐machine interface applications such as voice recognition (Figure 5h). During the test, distinctive words including “apple”, “peach”, “kiwi”, “banana”, and “passionfruit” were selected. The moisture sensor recorded the resistive changes during the pronunciation of these words. By analyzing factors such as the number of syllables and the positions of plosive sounds (e.g., “p”, “k”, and “b”), the sensor can distinguish between different words. Plosive sounds, also known as stop consonants, involve a complete closure of the vocal tract followed by a burst of air, which leads to a noticeable spike in humidity due to the sudden expulsion of breath. For instance, the “p” sound in “apple”, “peach”, and “passionfruit”, the “k” sound in “kiwi”, and the “b” sound in “banana” create distinct humidity patterns that the sensor can detect. These spikes in humidity caused by plosive sounds are unique markers that help in differentiating these words. The variations in humidity patterns associated with each word are influenced by the exhalation of breath during speech, which affects the absorption and desorption of water molecules in the GO film. Figure 5i shows the confusion matrix of voice classification using the SVM kernel model, which was trained with 10 sets of cross‐validation. The matrix indicates a classification accuracy of 100% for the five different voices. As such, each voice is perfectly distinguished without any misclassification, demonstrating the high effectiveness of the model and the sensor's ability to capture distinct resistance patterns for each voice.

## 3 Conclusion

In this work, we developed high‐performance AgNW networks through an innovative hydrochloric acid post‐treatment process. The method preferentially targets the surfaces and junctions of AgNWs, finely tuning the morphology of AgNWs. The resulting AgNWs show a thinner diameter with improved aspect ratio, increasing transparency from 62.6% to 76.7%, which makes them suitable for transparent applications. Benefiting from the well‐preserved network integrity and welding structure, enhanced electrical, heating, and mechanical properties are also demonstrated. A pressure sensor based on modified AgNWs shows better sensitivity than one with pristine AgNWs. By combining machine learning, the sensor can identify the pressing with different fingers, and a high accuracy of 94.5% is achieved with an SVM kernel at 80 iterations. Moreover, it correctly classifies the 26 letters of the alphabet via analysing the unique resistance patterns of their Morse code. The fabricated electrode is successfully integrated with a moisture‐sensitive GO layer for human respiration monitoring. In addition to recognizing human respiration behaviors (e.g., normal and deep breaths), the device is capable of detecting voices based on distinctive pronunciation patterns. The proposed strategy to design advanced AgNWs devices and further integration with machine learning provides a promising way for the development of smart and flexible electronics. In future studies, more comprehensive benchmarking of the sensor performance, including the evaluation of gauge factor, detection limit, long‐term cyclic stability, and hysteresis behavior, will be pursued to further validate its practical reliability in wearable sensing applications. In addition, the current machine learning model was trained using data collected from a single user under controlled conditions. Future work will focus on expanding the dataset to include multiuser inputs and performing inter‐subject validation across varied usage scenarios to enhance its robustness and generalizability for real‐world deployment.

## 4 Experimental Section

### Materials

Sodium chloride (NaCl), silver nitrate (AgNO_3_, ≥99%), ethylene glycol (EG), polyvinylpyrrolidone (PVP, molecular weight: 13 00 000), and nitric acid were ordered from Sigma–Aldrich. Hydrochloric acid (HCl, 32%) and acetic acid (glacial) were ordered from Chem‐Supply Pty Ltd, Australia. These chemicals were employed as received, without any additional purification.

### Synthesis of AgNWs

A modified method was employed for the synthesis of AgNWs,^[^ ^25^, ^30^ ^]^ requiring the sequential preparation of two unique mixtures. The first mixture was prepared by dissolving 1.112 g of PVP in 26.4 g of EG, which was subsequently heated and stirred to ensure even distribution. Then a second mixture was formulated by dissolving 0.179 g of AgNO_3_ in 15.9 g of EG. A solution containing 0.1 g of NaCl in 20 mL of EG was prepared, and 40 µL of it and 50 µL of acetic acid were added to the first mixture. After synthesis at 170 °C, the resultant solution was washed with ethanol by centrifuging. Finally, the cleaned NWs were dispersed in ethanol. For the fabrication of AgNWs‐based electrodes, a simple vacuum filtration method was used. The process began with thorough cleaning of the substrates using ethanol and subsequent UV treatment to eliminate surface organic contaminants. 1 mL of the original AgNWs solution and a membrane filter (0.45 µm pore size) were used for the filtration. The filtered films were then transferred onto the targeted substrates through a pressing technique. Then the obtained AgNWs electrodes were immersed in the 1 m HCl solution for etching.

### Preparation of Press Sensor and Moisture Sensor

The pressure sensor was fabricated by vacuum‐filtering AgNWs onto cured PDMS substrates. The PDMS was prepared by mixing 2 g of precursor with a 10:1 ratio of base to curing agent, pouring the mixture into a petri dish, and curing it at 70 °C for 1 h. The resulting AgNWs/PDMS film was then cut into 4 cm × 1 cm pieces. Two such films were laminated together to form the sandwich‐structured AgNWs/PDMS sensors, with both PDMS films serving as encapsulation layers. Copper tape was attached to both ends of the films using silver paste.^[^ ^66^ ^]^ Humidity sensors were prepared through dropping 50 µL of GO solution (0.5 mg mL^−1^) on cross‐finger‐patterned AgNWs on PI substrates. The fabricated electrodes were mounted on a 2.5 cm × 2.5 cm glass slide using double‐sided tape to protect the sensor from clamping damage during subsequent tests.^[^ ^30^ ^]^ HCl treatment of AgNWs improves their suitability for flexible device fabrication due to enhanced junction quality and network integrity.

### Characterization

The morphology and microstructure of the samples were investigated using a TEM (JEM‐F200, JEOL) and an FEI Nova NanoSEM 450 scanning electron microscope. For the characterization of crystalline structures, XRD analysis was utilized. Additionally, AFM was employed to investigate the surface topography and roughness. The optical property of the samples was determined using a PerkinElmer Lambda 365 spectrometer. FTIR (PerkinElmer) was used to investigate the functional groups of the GO film. The sheet resistance of the samples was studied with a four‐point probe resistance tester. The Electric Currents Module of COMSOL Multiphysics 6.1 under steady analysis mode was adopted for the finite‐element simulation of the electric potential distribution of AgNWs before and after melting. The bottom boundary, as the electric potential input terminal, was set to 3 V. The arc on the top is directly grounded (0 V). All initial electric potential value was 0 V. For the model where Ag nanowires were separated, the materials in the gap area were PDMS. Other parts were Ag. To illustrate the temperature distribution and visualize the thermal conduction behavior of AgNWs devices, was visualized using the Heat Transfer in Solids Module of COMSOL Multiphysics 6.1 under steady analysis mode for the finite‐element simulation. Considering the discrepancy of heat generation in different areas, some irregular areas were defined and were assumed to reach higher temperatures quickly according to experimental results. The temperatures of pristine and modified AgNWs were set as 174 and 277 °C, respectively. The temperature distribution in other areas derives from thermal conduction and self‐generated heat. The initial and ambient temperature of this module was 25 °C. In addition, the mechanical properties of the samples, particularly under stress from bending, were investigated via an FT2000 flexible electronics tester. This involved repeated bending tests (10 000 cycles) with a radius from 2.5 to 10 mm and a speed of 30°/s, flexing the electrodes from 0° to 90°. The human experiments were conducted on a participant (J. Fan, the first author) following the acquisition of his written consent. The resistance signals collected from pressure and humidity sensing experiments were processed for machine learning. A SVM kernel model was trained using MATLAB's Classification Learner toolkit. The input to the model consisted of time‐series resistance data collected under different stimuli. Through supervised learning, the SVM model identified characteristic patterns corresponding to specific signal types (e.g., pressure, humidity, or speech). In this way, machine learning serves as the decision‐making module that transforms raw sensor output into categorical recognition results. To evaluate model performance and mitigate overfitting, 10‐fold cross‐validation was applied. The dataset was automatically partitioned into 10 subsets, with each fold used iteratively as the validation set while the remaining data were used for training. The dataset used for training and evaluation was collected from a single user during a single session to demonstrate the feasibility of signal classification. Future studies will involve additional sessions and participants to assess reproducibility and generalizability.

## Conflict of Interest

The authors declare no conflict of interest.

## Supporting information



Supporting Information

## Data Availability

The data that support the findings of this study are available from the corresponding author upon reasonable request.

## References

[1] Y. Luo , M. R. Abidian , J.‐H. Ahn , D. Akinwande , A. M. Andrews , M. Antonietti , Z. Bao , M. Berggren , C. A. Berkey , C. J. Bettinger , ACS Nano 2023, 17, 5211. 36892156 10.1021/acsnano.2c12606 PMC11223676

[2] J. H. Lee , K. Cho , J. K. Kim , Adv. Mater. 2024, 36, 2310505. 10.1002/adma.202310505 38258951

[3] Y. Zhang , X. T. Zheng , X. Zhang , J. Pan , A. Voon‐Yew Thean , Chem. Rev. 2024, 124, 10386. 39189683 10.1021/acs.chemrev.3c00471

[4] C. Liu , Z. Feng , T. Yin , T. Wan , P. Guan , M. Li , L. Hu , C. H. Lin , Z. Han , H. Xu , W. Cheng , T. Wu , G. Liu , Y. Zhou , S. Peng , C. Wang , D. Chu , Adv. Mater. 2024, 36, 2403791. 10.1002/adma.202403791 38780429

[5] T. Sun , B. Feng , J. Huo , Y. Xiao , W. Wang , J. Peng , Z. Li , C. Du , W. Wang , G. Zou , L. Liu , Nano‐Micro Lett. 2024, 16, 14. 10.1007/s40820-023-01235-x PMC10643743 37955844

[6] M. Wang , T. Wang , Y. Luo , K. He , L. Pan , Z. Li , Z. Cui , Z. Liu , J. Tu , X. Chen , Adv. Funct. Mater. 2021, 31, 2008807.

[7] C. Krittanawong , A. J. Rogers , K. W. Johnson , Z. Wang , M. P. Turakhia , J. L. Halperin , S. M. Narayan , Nat. Rev. Cardiol. 2021, 18, 75. 33037325 10.1038/s41569-020-00445-9 PMC7545156

[8] N. Ha , K. Xu , G. Ren , A. Mitchell , J. Z. Ou , Adv. Intell. Syst. 2020, 2, 2000063.

[9] W. Song , Q. Ye , Z. Chen , J. Ge , L. Xie , Z. Ge , Adv. Mater. 2024, 36, 2311170. 10.1002/adma.202311170 38813892

[10] M. R. Azani , A. Hassanpour , T. Torres , Adv. Energy Mater. 2020, 10, 2002536.

[11] Z. Ma , X. Xiang , L. Shao , Y. Zhang , J. Gu , Angew. Chem., Int. Ed. 2022, 61, 202200705. 10.1002/anie.202200705 35122674

[12] Y. Ding , S. Xiong , L. Sun , Y. Wang , Y. Zhou , Y. Li , J. Peng , K. Fukuda , T. Someya , R. Liu , X. Zhang , Chem. Soc. Rev. 2024, 53, 7784. 38953906 10.1039/d4cs00080c

[13] Z. Wang , Y. Han , L. Yan , C. Gong , J. Kang , H. Zhang , X. Sun , L. Zhang , J. Lin , Q. Luo , C.‐Q. Ma , Adv. Funct. Mater. 2021, 31, 2007276.

[14] Y. Liu , X. Xu , Y. Wei , Y. Chen , M. Gao , Z. Zhang , C. Si , H. Li , X. Ji , J. Liang , Nano Lett. 2022, 22, 3784. 35486490 10.1021/acs.nanolett.2c00876

[15] T. Wan , P. Guan , X. Guan , L. Hu , T. Wu , C. Cazorla , D. Chu , ACS Appl. Mater. Interfaces 2020, 12, 34086. 32643927 10.1021/acsami.0c07950

[16] Y. Zhao , X. Wang , S. Yang , E. Kuttner , A. A. Taylor , R. Salemmilani , X. Liu , M. Moskovits , B. Wu , A. Dehestani , J. F. Li , M. F. Chisholm , Z. Q. Tian , F. R. Fan , J. Jiang , G. D. Stucky , J. Am. Chem. Soc. 2019, 141, 13977. 31436416 10.1021/jacs.9b07172

[17] Y. Roh , S. Lee , S. M. Won , S. Hwang , D. Gong , C. Kim , I. Hong , D. Lim , H. Kim , M. Kim , B. Kim , T. Kim , S. Im , D. Shin , U. Kim , J. Choi , J.‐S. Koh , D. Kang , S. Han , Nat. Electron. 2024, 7, 66.

[18] Y. Liu , Z. Xu , X. Ji , X. Xu , F. Chen , X. Pan , Z. Fu , Y. Chen , Z. Zhang , H. Liu , B. Cheng , J. Liang , Nat. Commun. 2024, 15, 5354. 38918424 10.1038/s41467-024-49787-9 PMC11200319

[19] P. Guan , R. Zhu , Y. Zhu , F. Chen , T. Wan , Z. Xu , R. Joshi , Z. Han , L. Hu , T. Wu , Y. Lu , D. Chu , Crit. Rev. Solid State Mater. Sci. 2022, 47, 435.

[20] J. Han , J. Yang , W. Gao , H. Bai , Adv. Funct. Mater. 2021, 31, 2010155.

[21] C. Gabbett , A. G. Kelly , E. Coleman , L. Doolan , T. Carey , K. Synnatschke , S. Liu , A. Dawson , D. O'Suilleabhain , J. Munuera , E. Caffrey , J. B. Boland , Z. Sofer , G. Ghosh , S. Kinge , L. D. A. Siebbeles , N. Yadav , J. K. Vij , M. A. Aslam , A. Matkovic , J. N. Coleman , Nat. Commun. 2024, 15, 4517. 38806479 10.1038/s41467-024-48614-5 PMC11133347

[22] W. Zhao , J. Dong , Z. Li , B. Zhou , C. Liu , Y. Feng , Adv. Sci. 2024, 11, 2406758. 10.1002/advs.202406758 PMC11481190 39116320

[23] K. Zhang , L. Meng , M. Zhang , Y. Li , L. Jiang , H. Liu , Adv. Funct. Mater. 2024, 34, 2308468.

[24] J. Yang , L. Chang , H. Zhao , X. Zhang , Z. Cao , L. J. I. Jiang , InfoMat 2024, 6, 12529.

[25] Y. Zhu , T. Wan , P. Guan , Y. Wang , T. Wu , Z. Han , G. Tang , D. Chu , J. Colloid Interface Sci. 2020, 566, 375. 32018177 10.1016/j.jcis.2020.01.111

[26] S. Huang , Y. Liu , M. Jafari , M. Siaj , H. Wang , S. Xiao , D. Ma , Adv. Funct. Mater. 2021, 31, 2010022.

[27] W. Chen , L.‐X. Liu , H.‐B. Zhang , Z.‐Z. Yu , ACS Nano 2020, 14, 16643. 32453550 10.1021/acsnano.0c01635

[28] E. Lee , J. Ahn , H. C. Kwon , S. Ma , K. Kim , S. Yun , J. Moon , Adv. Energy Mater. 2018, 8, 1702182.

[29] S. Yu , X. Liu , P. Yang , L. Zhao , H. Dong , C. Wu , X. Li , J. Xiong , Chem. Eng. J. 2022, 446, 137481.

[30] J. Fan , T. Wan , Y. He , C. Liu , T. Mei , Y. C. Kuo , Z. Feng , P. Guan , C. H. Lin , M. Li , Small Struct. 2024, 5, 2400208.

[31] L. T. Trinh , T. T. L. To , P. Ko , K. Woo , S. Kwon , J. Rho , H. Youn , Small 2024, 20, 2403702. 10.1002/smll.202403702 39087377

[32] J. J. Patil , M. L. Reese , E. Lee , J. C. Grossman , ACS Appl. Mater. Interfaces 2022, 14, 4423. 35029366 10.1021/acsami.1c20521

[33] X. Ren , X. Mei , J. Zhou , X. Wang , F. Wei , H. Mei , S. Zhao , Y. Lu , J. Cui , Adv. Mater. 2024, 36, 2408575. 10.1002/adma.202408575 39400396

[34] E. C. Garnett , W. Cai , J. J. Cha , F. Mahmood , S. T. Connor , M. Greyson Christoforo , Y. Cui , M. D. McGehee , M. L. Brongersma , Nat. Mater. 2012, 11, 241. 22306769 10.1038/nmat3238

[35] M. Du , Z. Yang , Y. Miao , C. Wang , P. Dong , H. Wang , K. Guo , Adv. Funct. Mater. 2024, 34, 2404567.

[36] J. Y. Tseng , L. Lee , Y. C. Huang , J. H. Chang , T. Y. Su , Y. C. Shih , H. W. Lin , Y. L. Chueh , Small 2018, 14, 1800541. 10.1002/smll.201800541 30133161

[37] B. Hwang , H. A. S. Shin , T. Kim , Y. C. Joo , S. M. Han , Small 2014, 10, 3397. 24789010 10.1002/smll.201303906

[38] M. Lagrange , D. P. Langley , G. Giusti , C. Jimenez , Y. Brechet , D. Bellet , Nanoscale 2015, 7, 17410. 26437607 10.1039/c5nr04084a

[39] J. Lee , I. Lee , T. S. Kim , J. Y. Lee , Small 2013, 9, 2887. 23606676 10.1002/smll.201203142

[40] G. Zeng , W. Chen , X. Chen , Y. Hu , Y. Chen , B. Zhang , H. Chen , W. Sun , Y. Shen , Y. Li , J. Am. Chem. Soc. 2022, 144, 8658. 35469397 10.1021/jacs.2c01503

[41] H. Kang , Y. Kim , S. Cheon , G. R. Yi , J. H. Cho , ACS Appl. Mater. Interfaces 2017, 9, 30779. 28820234 10.1021/acsami.7b09839

[42] O. Cakir , D. Doganay , M. Cugunlular , M. O. Cicek , O. Demircioglu , S. Coskun , H. E. Unalan , Nano Energy 2024, 128, 109940.

[43] S.‐S. Yoon , D.‐Y. Khang , Nano Lett. 2016, 16, 3550. 27159354 10.1021/acs.nanolett.6b00621

[44] X. Liang , T. Zhao , P. Zhu , Y. Hu , R. Sun , C. P. Wong , ACS Appl. Mater. Interfaces 2017, 9, 40857. 29125737 10.1021/acsami.7b13048

[45] S. Bai , H. Wang , H. Yang , H. Zhang , X. Guo , Mater. Res. Express 2018, 5, 026406.

[46] J. Yang , Y. Zhou , S. Du , T. Zhou , Y. Wang , Langmuir 2024, 40, 16384. 39051492 10.1021/acs.langmuir.4c01631

[47] J. Gu , X. Wang , H. Chen , S. Yang , H. Feng , X. Ma , H. Ji , J. Wei , M. Li , Nanotechnology 2018, 29, 265703. 29620018 10.1088/1361-6528/aabbbc

[48] C. M. Cobley , M. Rycenga , F. Zhou , Z.‐Y. Li , Y. Xia , J. Phys. Chem. C 2009, 113, 16975.

[49] H. J. Lee , S. Oh , K. Y. Cho , W. L. Jeong , D. S. Lee , S. J. Park , ACS Appl. Mater. Interfaces 2018, 10, 14124. 29620842 10.1021/acsami.8b00837

[50] Z. R. Zhu , W. Geng , Q. Zhu , A. S. Ethiraj , T. Wang , L. C. Jing , Y. J. Ning , Y. Tian , W. H. Geng , L. Wu , H. Z. Geng , Nanotechnology 2021, 32, 015708. 32937609 10.1088/1361-6528/abb906

[51] H. Liu , J. Wu , Y. Fu , B. Wang , Q. Yang , G. D. Sharma , M. L. Keshtov , Z. Xie , Thin Solid Films 2021, 718, 138486.

[52] T. Sannicolo , N. Charvin , L. Flandin , S. Kraus , D. T. Papanastasiou , C. Celle , J. P. Simonato , D. Munoz‐Rojas , C. Jimenez , D. Bellet , ACS Nano 2018, 12, 4648. 29722956 10.1021/acsnano.8b01242

[53] J. Fan , Y.‐C. Kuo , T. Yin , P. Guan , L. Meng , F. Chen , Z. Feng , C. Liu , T. Wan , Z. Han , L. Hu , S. Peng , T. Wu , D. Chu , ACS Appl. Mater. Interfaces 2024, 16, 39600. 39041667 10.1021/acsami.4c06483

[54] Y. Ge , X. Duan , M. Zhang , L. Mei , J. Hu , W. Hu , X. Duan , J. Am. Chem. Soc. 2018, 140, 193. 29185776 10.1021/jacs.7b07851

[55] V.‐T. Nguyen , G. A. V. Phan , Mater. Today Commun. 2023, 34, 105051.

[56] M. Bian , Y. Qian , H. Cao , T. Huang , Z. Ren , X. Dai , S. Zhang , Y. Qiu , R. Si , L. Yang , S. Yin , ACS Appl. Mater. Interfaces 2023, 15, 13307. 36880523 10.1021/acsami.2c21996

[57] G. Zeng , W. Chen , X. Chen , Y. Hu , Y. Chen , B. Zhang , H. Chen , W. Sun , Y. Shen , Y. Li , F. Yan , Y. Li , J. Am Chem. Soc. 2022, 144, 8658. 35469397 10.1021/jacs.2c01503

[58] Y. He , H. Ni , D. Mishra , S. Peng , H.‐P. Phan , C. Boyer , C. H. Wang , J. Zhang , Nano Energy 2024, 127, 109737.

[59] N. T. Beigh , F. T. Beigh , D. Mallick , Nano Energy 2023, 116, 108824.

[60] A. Patle , D. S. Chouhan , 2013 Int. Conf. on Advances in Technology and Engineering (ICATE) , Mumbai, India, January 2013.

[61] J. H. Lee , J. S. Heo , Y. J. Kim , J. Eom , H. J. Jung , J. W. Kim , I. Kim , H. H. Park , H. S. Mo , Y. H. Kim , S. K. Park , Adv. Mater 2020, 32, 2000969. 10.1002/adma.202000969 32310332

[62] R. Zhu , Y. Zhu , F. Chen , R. Patterson , Y. Zhou , T. Wan , L. Hu , T. Wu , R. Joshi , M. Li , C. Cazorla , Y. Lu , Z. Han , D. Chu , Nano Energy 2022, 94, 106942.

[63] Y. Wang , L. Zhang , Z. Zhang , P. Sun , H. Chen , Langmuir 2020, 36, 9443. 32693594 10.1021/acs.langmuir.0c01315

[64] N. Wan , T. Wang , X.‐y. Tan , S. Lu , L.‐l. Zhou , J.‐q. Huang , W. Pan , Y.‐m. Yang , Z.‐y. Shao , J. Phys. Chem. C 2018, 122, 830.

[65] R. Alrammouz , J. Podlecki , A. Vena , R. Garcia , P. Abboud , R. Habchi , B. Sorli , Sens. Actuators, B 2019, 298, 126892.

[66] J. Lee , G. Kim , D.‐K. Shin , J. Park , IEEE Sens. J. 2018, 18, 9919

