# Supplementary material for: Thinned and Welded Silver Nanowires for Intelligent Pressure and Humidity Sensing Enabled by Machine Learning
Source: Adv Sci (Weinh). 2025 Aug 27;12(43):e07610. doi: 10.1002/advs.202507610 (PMC12631923; doi:10.1002/advs.202507610)
Supplement: Supplementary file 1 — Supporting Information [file ADVS-12-e07610-s001.docx]

Supporting Information

**Thinned and Welded Silver Nanowires for Intelligent Pressure and Humidity Sensing Enabled by Machine Learning**

Jiajun Fan, Tao Wan^*^, Tao Yin, Ziheng Feng, Peiyuan Guan, Tianxu Huang, Chao Liu^*^, Mengyao Li^*^, Shuying Wu, Shuhua Peng^*^, Shihao Huang, Zhaojun Han, Dongchen Qi, Wenlong Cheng, Dewei Chu

J. Fan, T. Wan, T. Yin, Z. Feng, P. Guan, T. Huang, C. Liu, M. Li, S. Huang, D. Chu

School of Materials Science and Engineering, The University of New South Wales, Sydney, NSW, 2052, Australia

E-mail: tao.wan@unsw.edu.au; chao.liu4@unsw.edu.au; mengyao.li1@unsw.edu.au

S. Wu

School of Aerospace, Mechanical and Mechatronic Engineering, The University of Sydney, Sydney, New South Wales 2006, Australia

S. Peng

School of Mechanical Engineering, The University of New South Wales, Sydney, NSW 2052, Australia

E-mail: shuhua.peng@unsw.edu.au

Z. Han

Eastern Institute of Technology, Ningbo, Zhejiang 315200, China

School of Mechanical, Medical and Process Engineering, Queensland University of Technology, Brisbane, QLD 4000, Australia

School of Chemical Engineering, The University of New South Wales, Sydney, NSW, 2052, Australia

D. Qi

School of Chemistry and Physics, Queensland University of Technology, Brisbane QLD 4000, Australia

Centre for Materials Science, Queensland University of Technology, Brisbane QLD 4000, Australia

W. Cheng

School of Biomedical Engineering, The University of Sydney, Darlington, New South Wales 2008, Australia


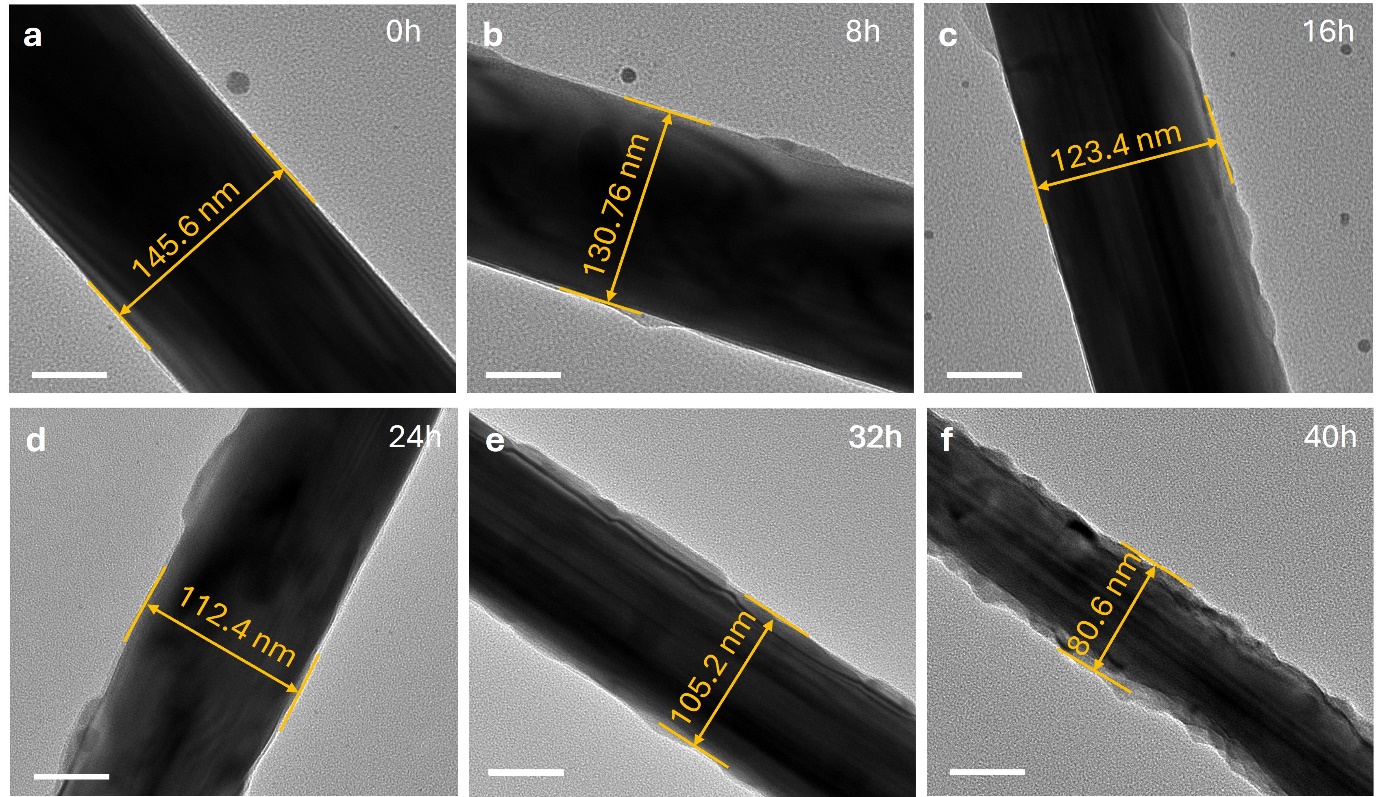


**Figure S1.** TEM images of AgNWs through HCl treatment: (a) 0h; (b) 8h; (c) 16h; (d) 24h; (e) 32h; (f) 40h. Scale bar: 50 nm.


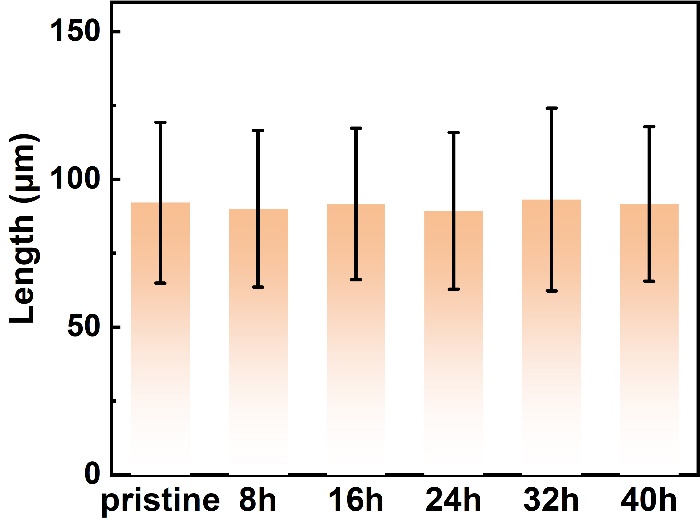


**Figure S2.** Average lengths of pristine and HCl-treated AgNWs.


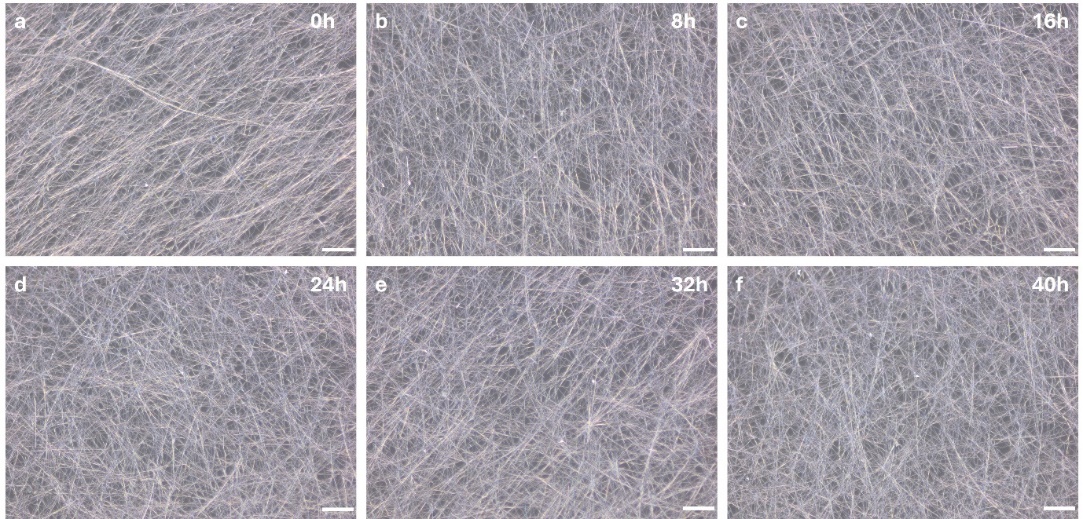


**Figure S3.** Optical images of AgNWs after 1M HCl post treatment from 0h to 40h: (a) 0h; (b) 8h; (c) 16h; (d) 24h; (e) 32h; (f) 40h. Scale bar: 20 µm.


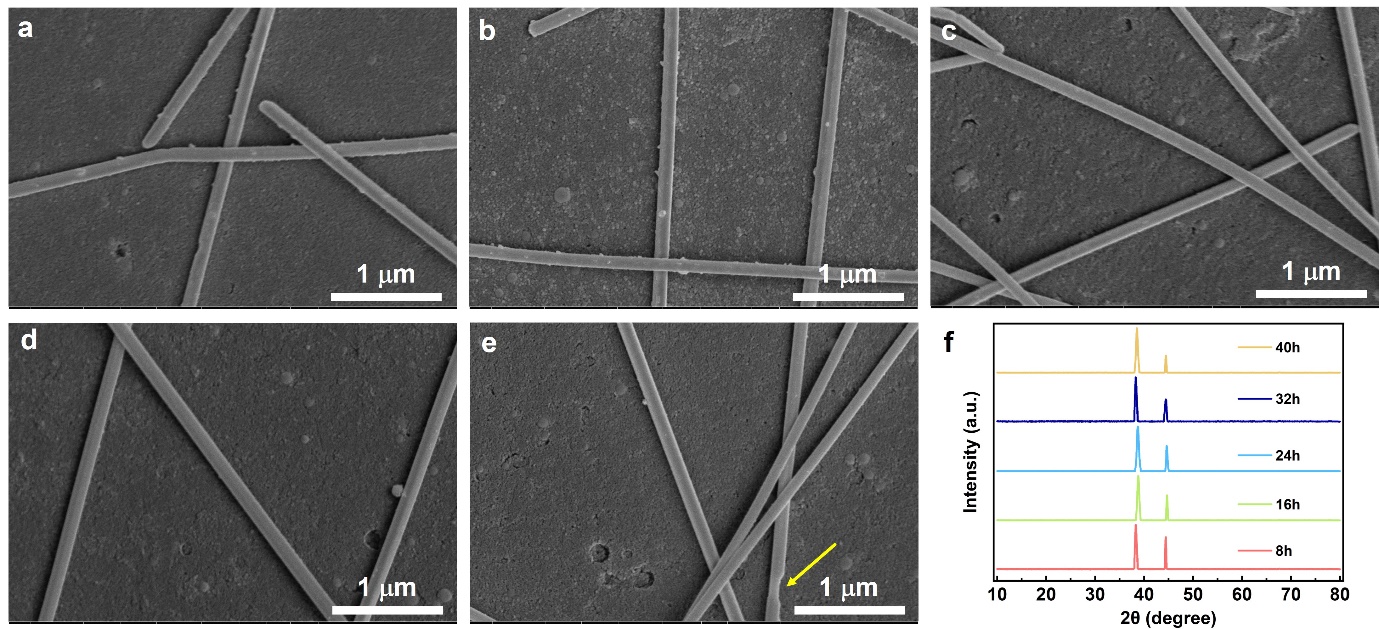


**Figure S4.** (a-e) SEM images of AgNWs after 1M acetic acid post treatment from 8 h to 40 h. (f) XRD patterns of AgNWs corresponding to the varied post treatment time. Unlike the HCl treatment, AgNWs are randomly etched or corroded in the acetic acid condition, indicated by the yellow arrow.


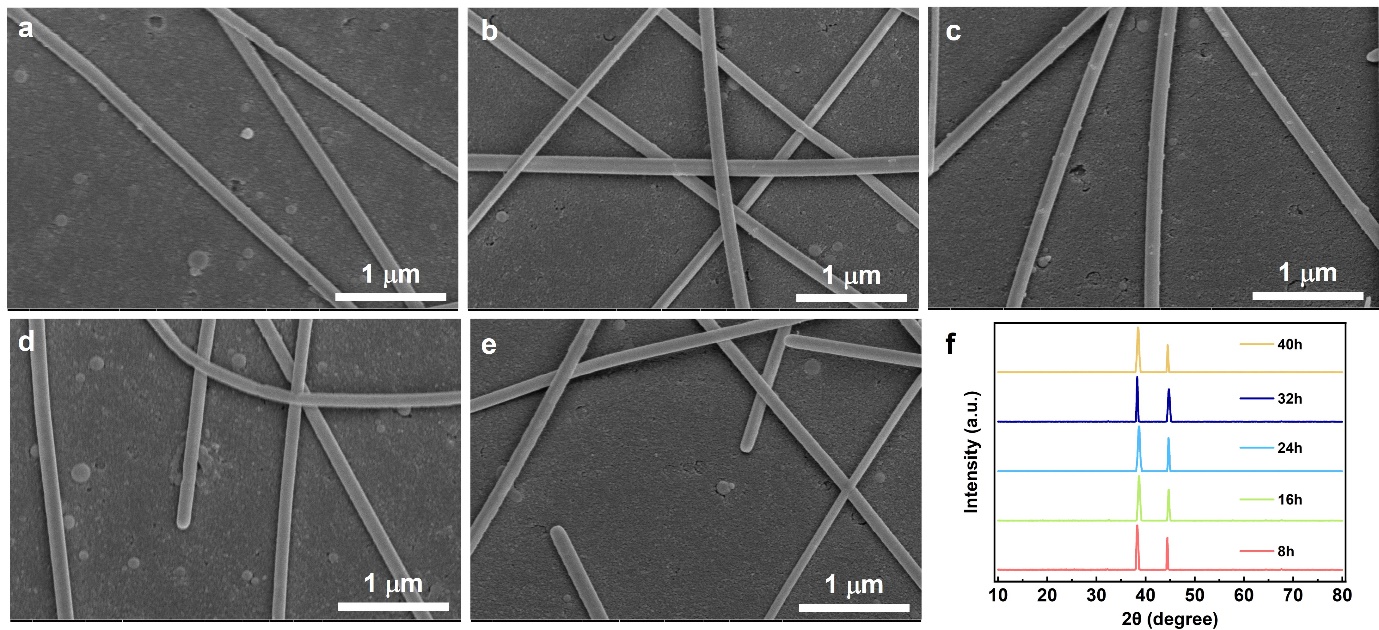


**Figure S5.** (a-e) SEM images of AgNWs after 1M nitric acid post treatment from 8h to 40h. (f) XRD patterns of the AgNWs corresponding to the varied post treatment time. Although nitric acid is known for its strong oxidizing properties, it will undergo photodecomposition when exposed to light. Therefore, the concentration of nitric acid available to react with AgNWs could be significantly reduced, and accordingly most NWs remain unaffected due to an insufficiently etching environment.


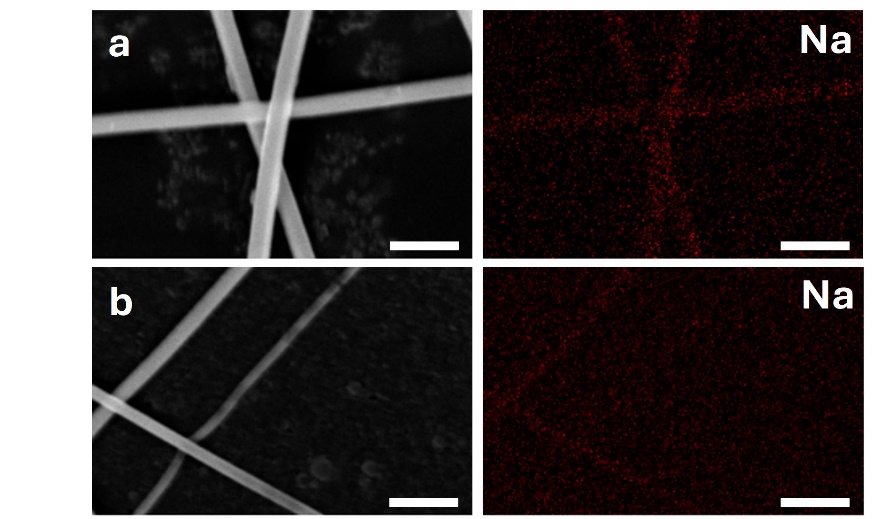


**Figure S6.** SEM and EDS element mapping of (a) pristine and (b) HCl-treated AgNWs. Scale bar: 500 nm.


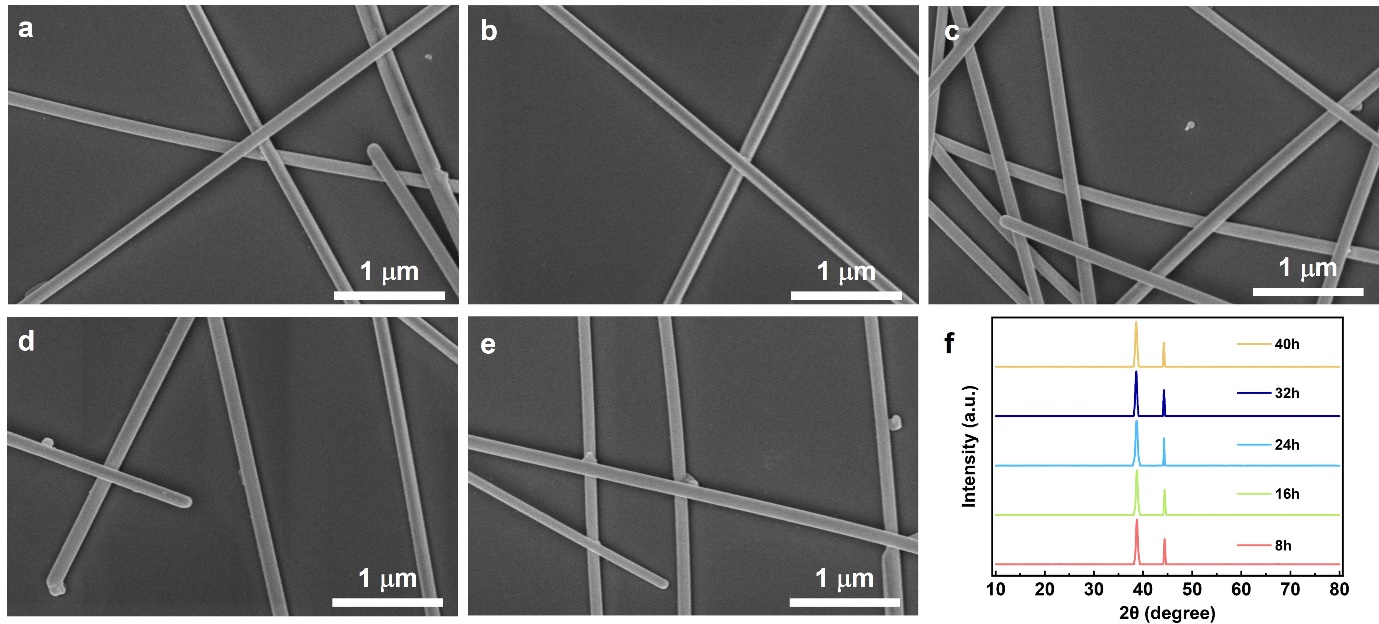


**Figure S7.** (a-e) SEM images of AgNWs after 0.1M HCl post treatment from 8h to 40h. (f) XRD patterns of the AgNWs corresponding to the varied post treatment time.


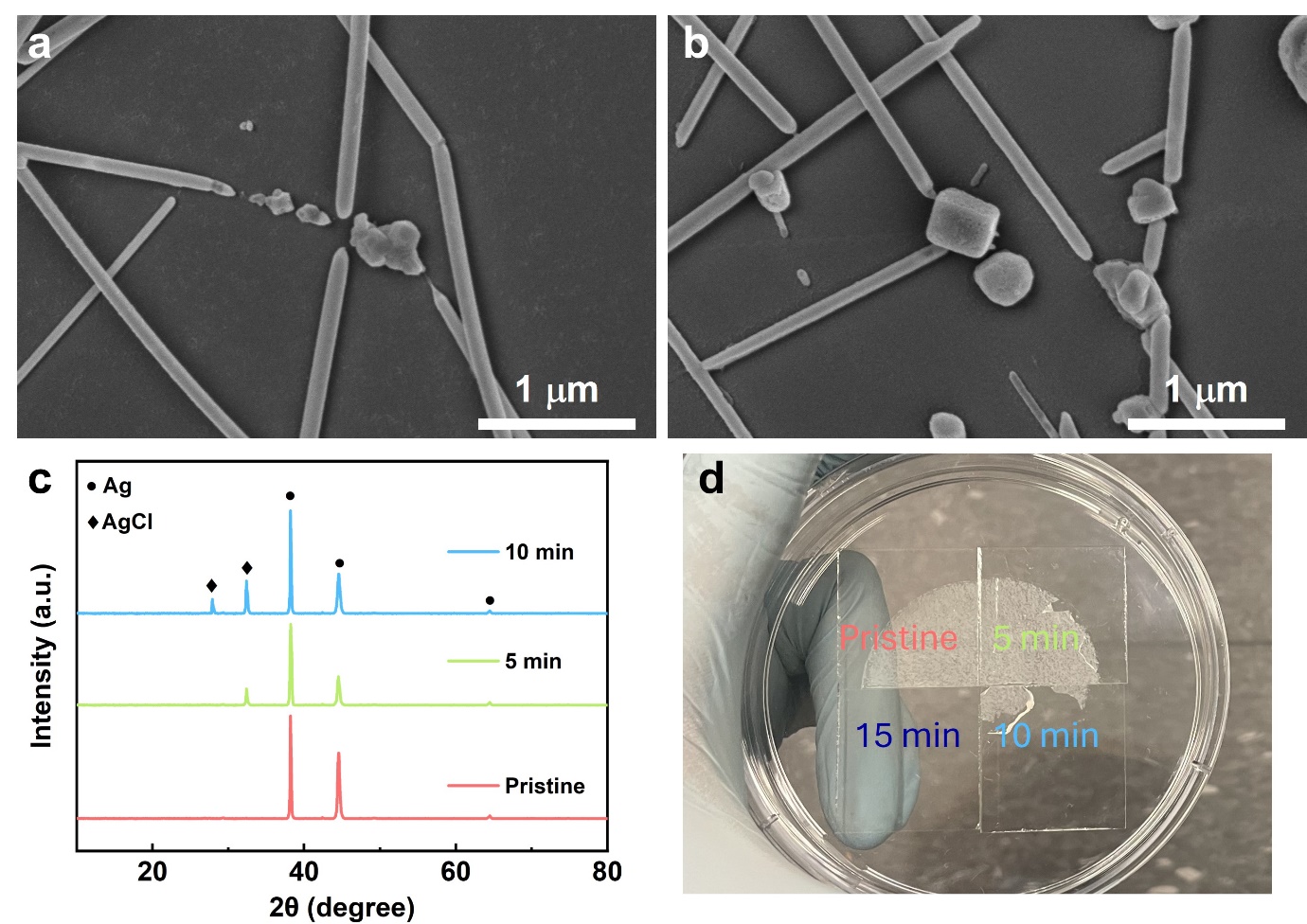


**Figure S8.** (a, b) SEM images of AgNWs treated with 32 wt% HCl: (a) 5 min and (b) 10 min. (c) XRD patterns of AgNWs corresponding to the varied post treatment time. (d) Photograph of AgNWs corresponding to different immersion time.


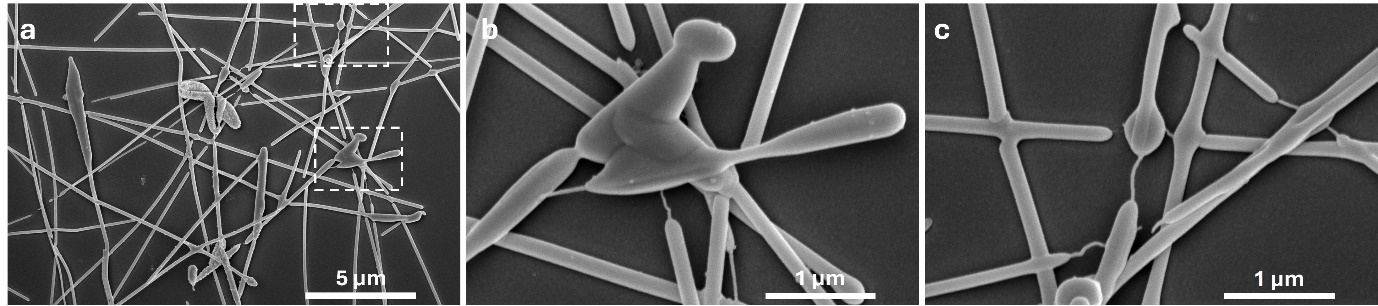


**Figure S9.** SEM images of AgNWs after electrical failure.


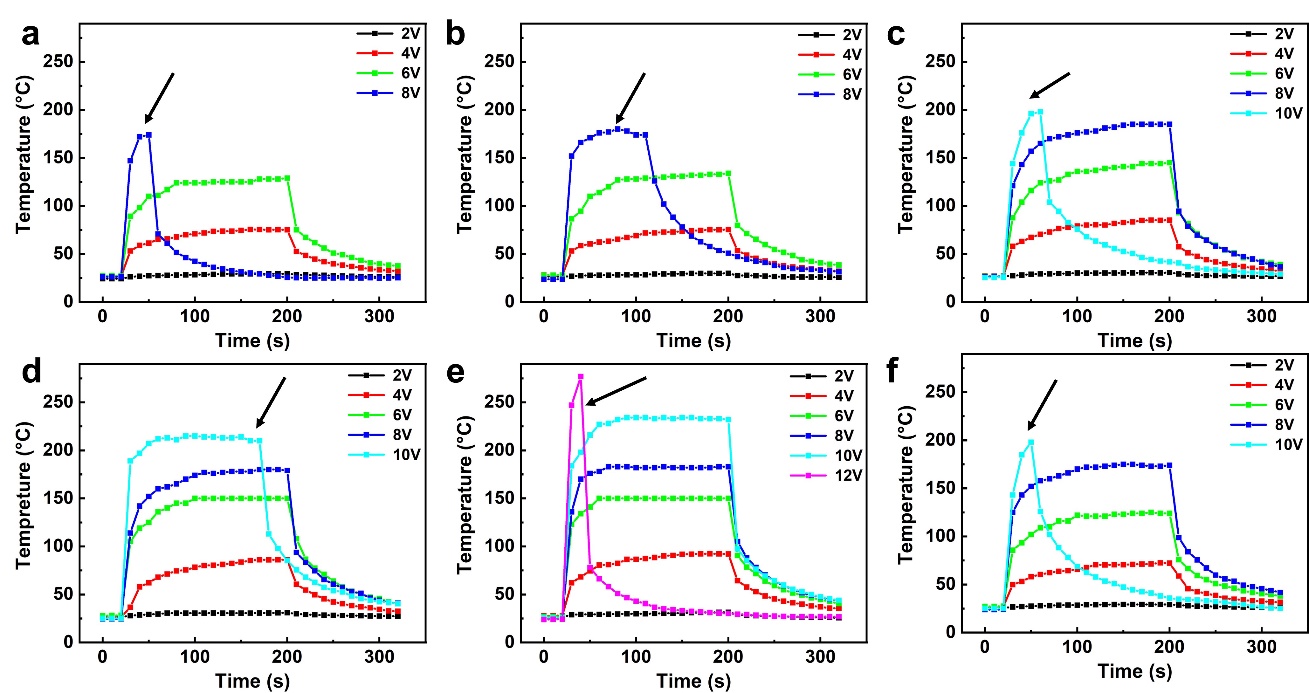


**Figure S10.** Heating performance of (a) pristine AgNWs and acid-treated AgNWs: (b) 8h, (c) 16h, (d) 24h, (e) 32h, and (f) 40h. Device failure is indicated by the arrow.


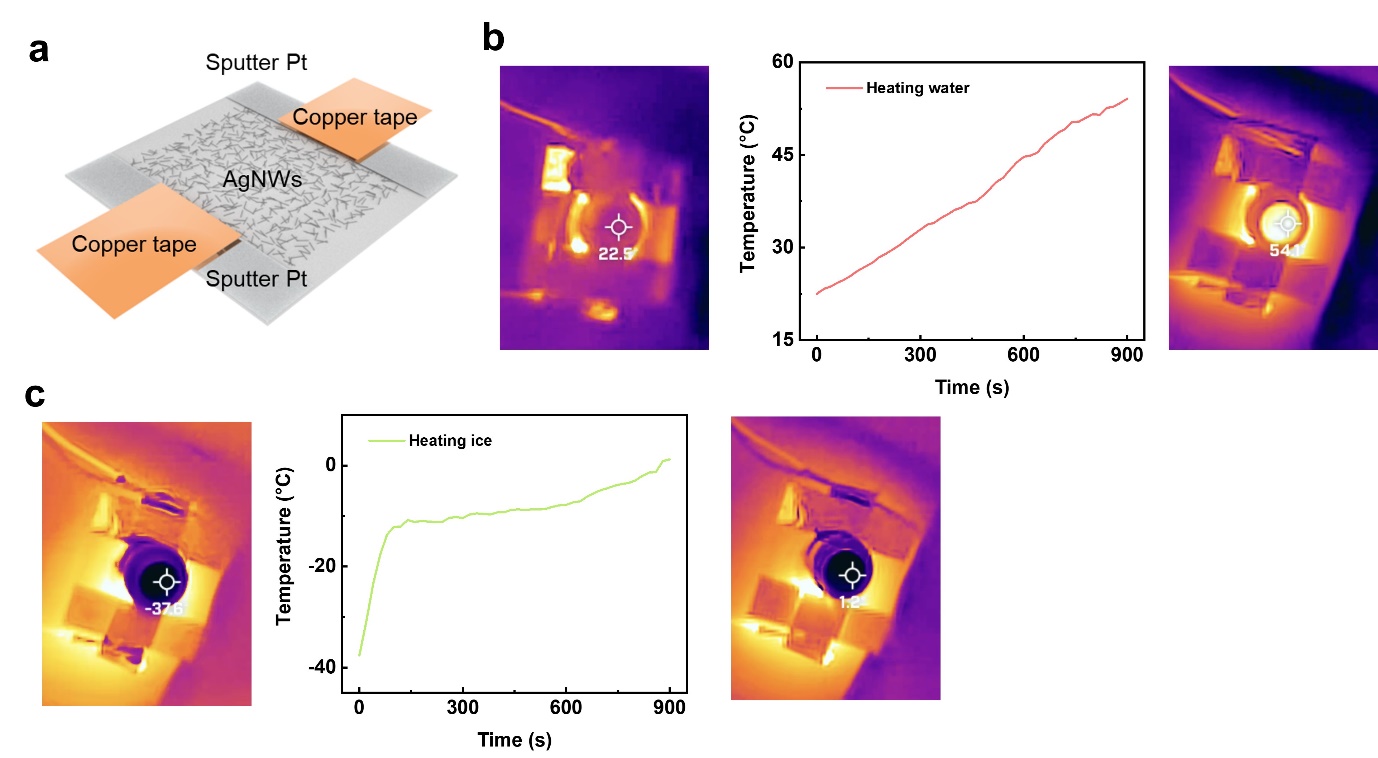


**Figure S11.** (a) Schematic of an AgNWs heater. Heating curves and the corresponding Infrared images: (b) heating water and (c) heating ice.


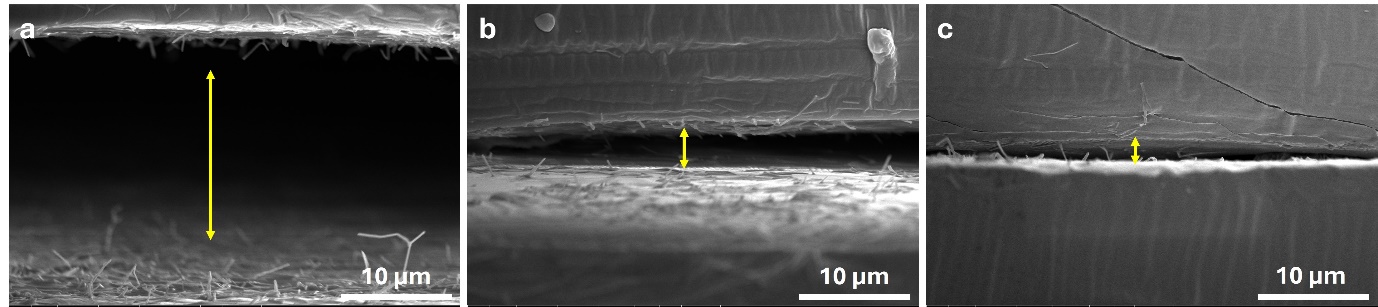


**Figure S12.** SEM images of two AgNWs films with varying contact conditions.


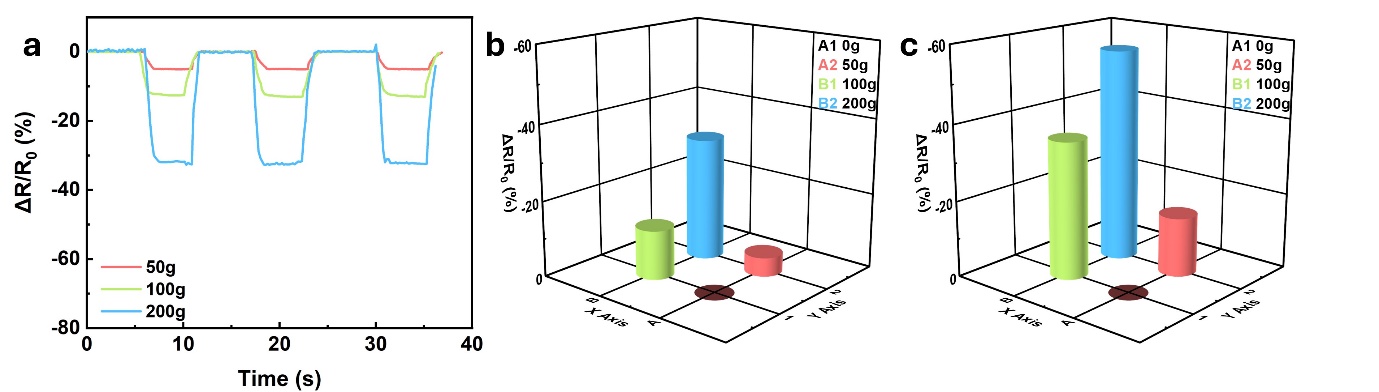


**Figure S13.** Relative resistance changes induced by different weights applied to sensors based on (a, b) pristine and (c) HCl-treated AgNWs.


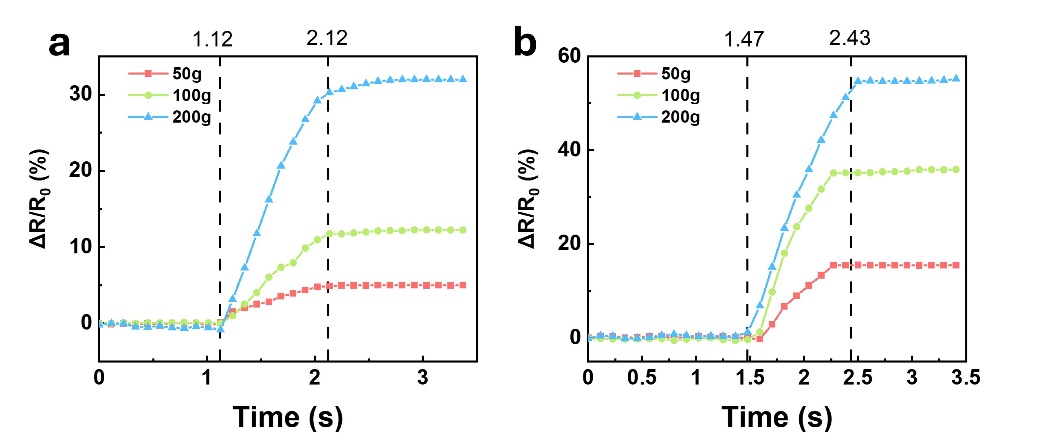


**Figure S14.** Relative resistance changes over time under different weights applied to the sensor fabricated from AgNWs treated with HCl for 32h.


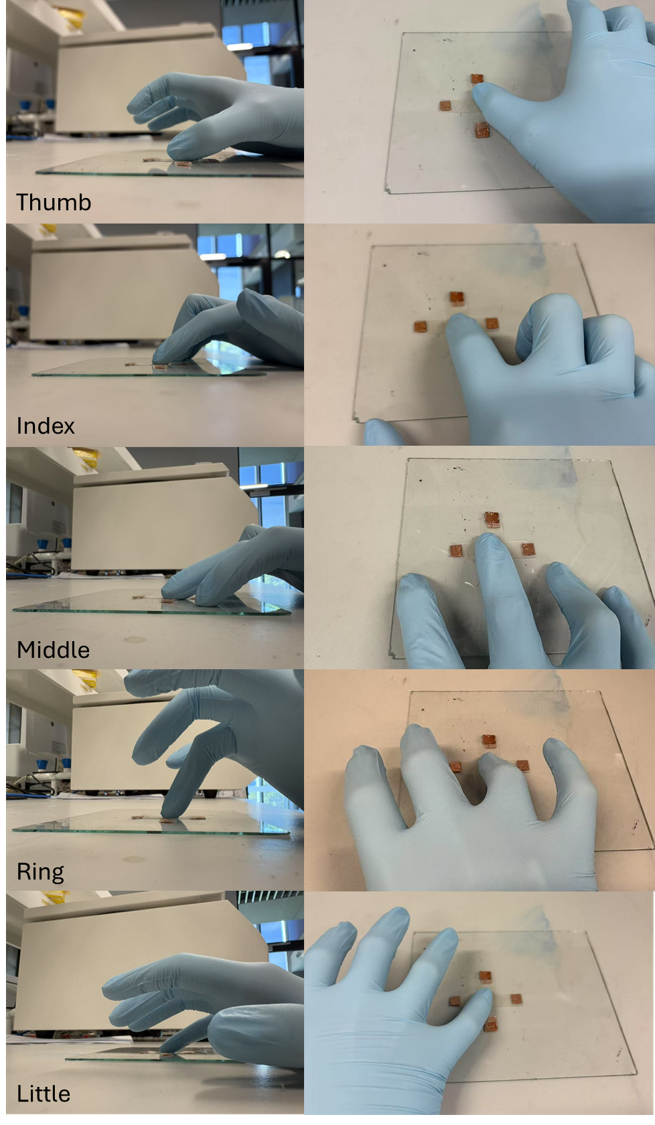


**Figure S15.** Photographs illustrating how each finger interacts with the pressure sensor during pressing.


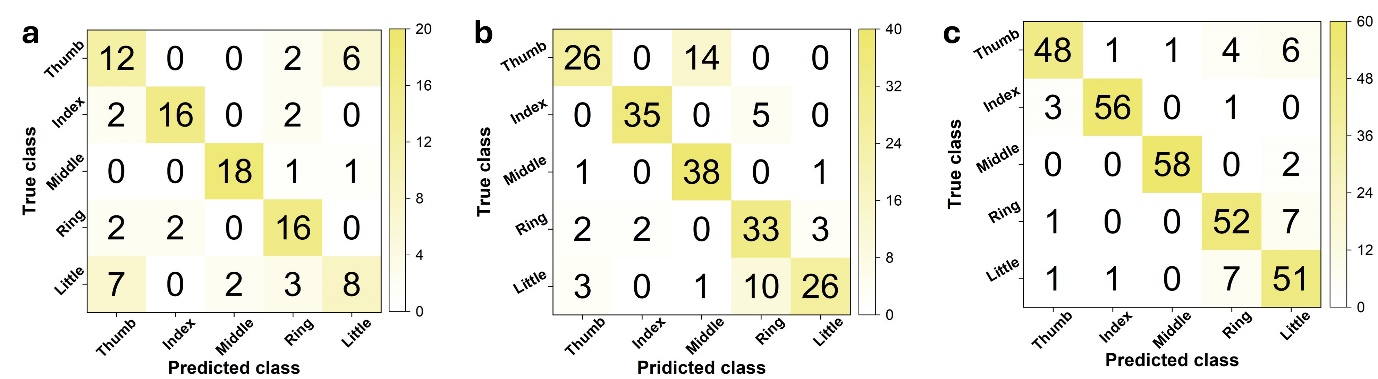


**Figure S16.** Confusion matrices showing classification accuracy after (a) 20 training iterations, (b) 40 training iterations, and (c) 60 training iterations.


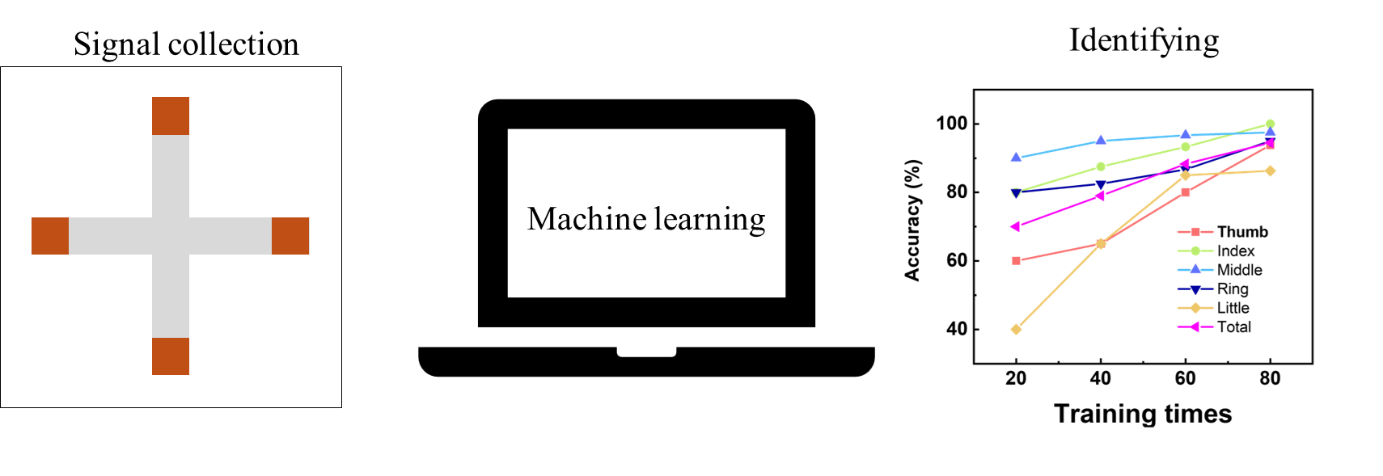


**Figure S17.** Schematic illustration of the signal recognition process based on AgNW sensor and machine learning. Pressing signals are classified by a trained support vector machine (SVM) model, distinct with five different fingers (thumb, index, middle, ring, and little) across various training iterations (20, 40, 60, and 80).


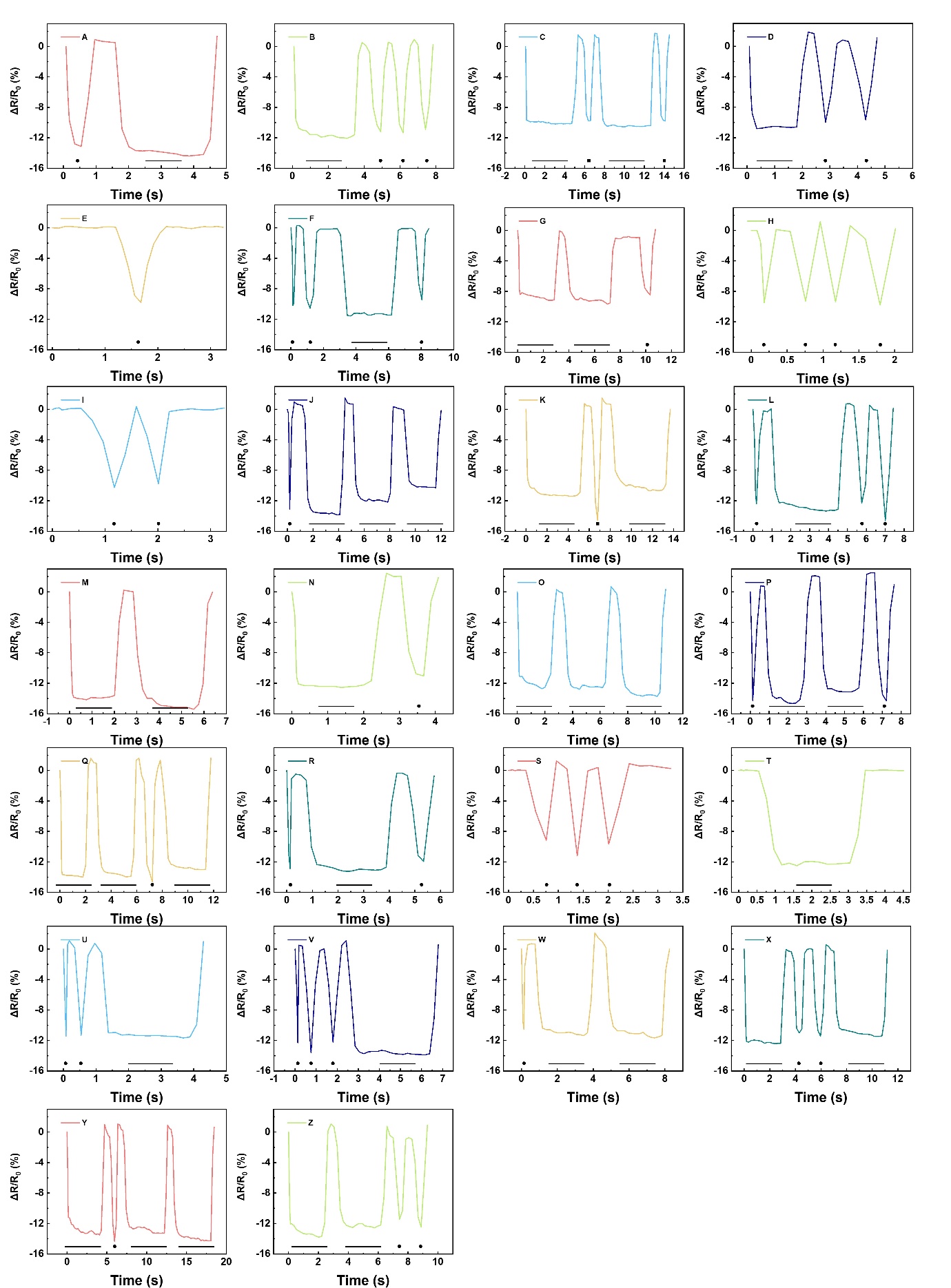


**Figure S18.** Relative resistance changes of the sensor representing the Morse code of the 26 letters of the alphabet.


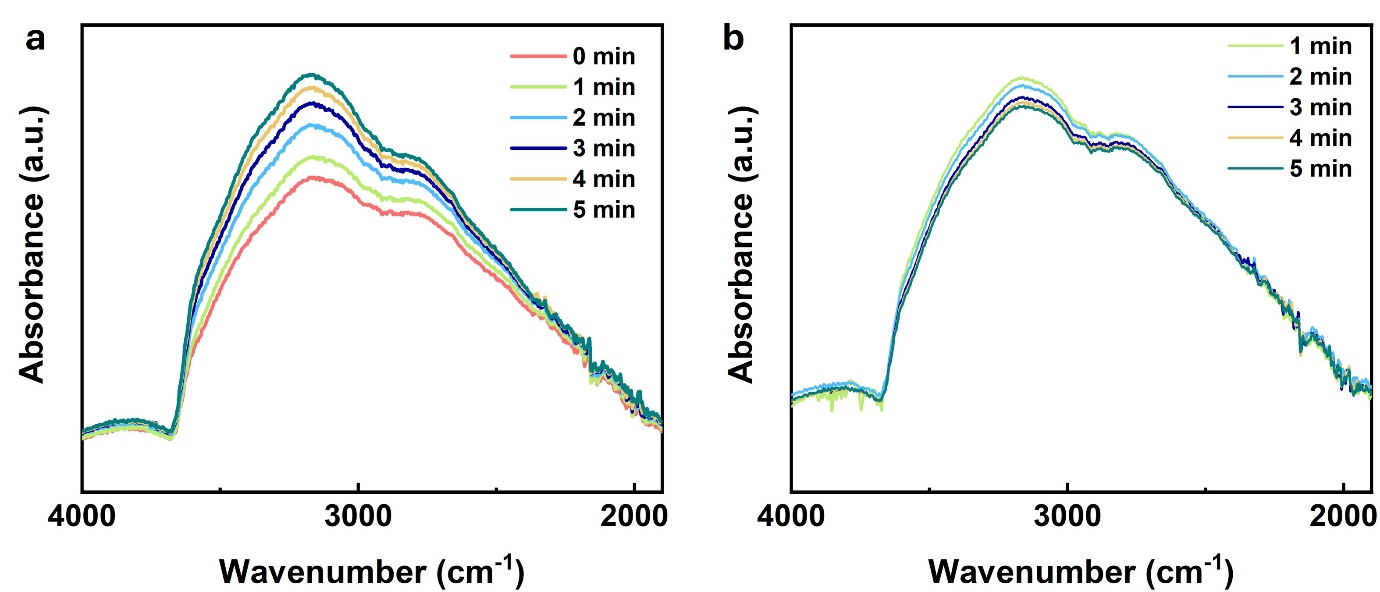


**Figure S19.** The FTIR spectra of GO film as a function of time: (a) moisture exposure and (b) dry N_2_ blowing.


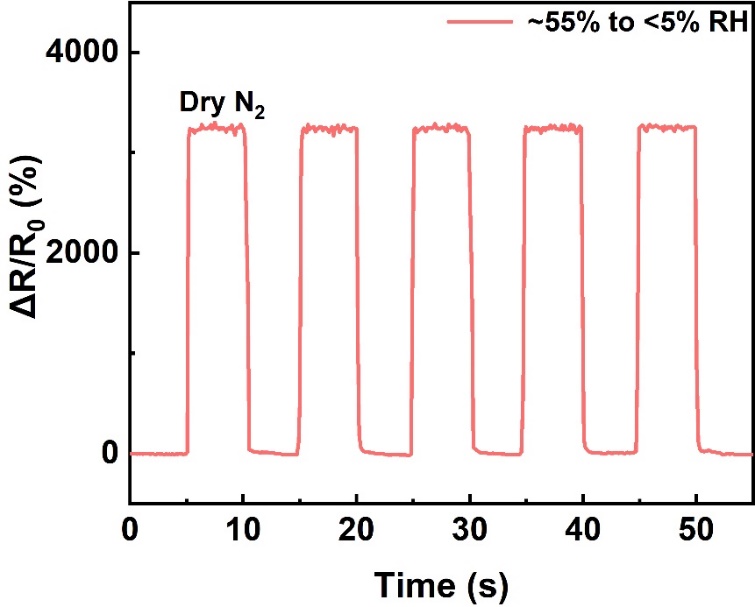


**Figure S20.** Relative resistance change of the sensor under different humidities controlled dry N_2_.

Table S1. Comparison of different sensors with machine learning utilized for signal recognition.

|  | **Sensors** | **Materials** | **Classifier** | **Iterations** | **Accuracy** |
| --- | --- | --- | --- | --- | --- |
| This work | Pressure sensors | AgNWs | Support Vector Machine Kernel | 80 | 94.5% |
| [1] | Pressure sensors | MoS_2_/HEC/PU | Support Vector Machine | 350 | 97.15% |
| [2] | Pressure sensors | MXene and graphene  nanoribbon | Artificial Neural Network | 180 | 97.2% |
| [3] | Pressure sensors | Commercial pressure sensor | Random Forest | 312587 | 98.82% |
| [4] | Pressure sensors | Cross-striped nanocarbon | Neural Network | 250 | 98.9% |
| [5] | Pressure sensors | Barium Titanate/SU-8 nanocomposite | 2D Convolutional Neural Network | 311950 | 98.5% |
| [6] | Pressure sensors | MXene coated wrinkle structure | Extreme Gradient Boosting | 50 | 95 ± 3% |
| [7] | Strain sensors | MXene-single walled carbon nanotube-PVA | Artificial Neural Network | Not provided | 100% |
| [8] | Biosensors | Silver ink | Hyperdimensional Computing, | 1 | 97.12% |
| [9] | Triboelectric sensors | Polystyrene/MoS_2_-Poly(vinylidene fluoride-co-hexafluoropropylene) | Bagged Tree | 98 | 86% |
| [10] | laryngeal sensors | 2-(Methacryloyloxy)ethyl]dimethyl-(3-sulfopropyl) ammonium hydroxide (DMAPS) | Convolutional Neural Network | 100 | 98.2% |
| [11] | Stretchable sensors | Graphene/Polyimide nanomembrane | Convolutional Neural Network | 100 | 94.54% |
| [12] | Acoustic sensors | Lead-Zirconate-Titanate (PZT) Thin Film | Gaussian Mixture Model | 150 | 90% |

**References**

[1] X. Chen, D. Zhang, H. Luan, C. Yang, W. Yan, W. Liu, Flexible Pressure Sensors Based on Molybdenum Disulfide/Hydroxyethyl Cellulose/Polyurethane Sponge for Motion Detection and Speech Recognition Using Machine Learning, ACS Appl Mater Interfaces 15(1) (2023) 2043-2053. <https://doi.org/10.1021/acsami.2c16730>.

[2] H.J. Lee, J.C. Yang, J. Choi, J. Kim, G.S. Lee, S.P. Sasikala, G.H. Lee, S.K. Park, H.M. Lee, J.Y. Sim, S. Park, S.O. Kim, Hetero-Dimensional 2D Ti(3)C(2)T(x) MXene and 1D Graphene Nanoribbon Hybrids for Machine Learning-Assisted Pressure Sensors, ACS Nano 15(6) (2021) 10347-10356. <https://doi.org/10.1021/acsnano.1c02567>.

[3] K. Bourahmoune, K. Ishac, T. Amagasa, Intelligent Posture Training: Machine-Learning-Powered Human Sitting Posture Recognition Based on a Pressure-Sensing IoT Cushion, Sensors (Basel) 22(14) (2022). <https://doi.org/10.3390/s22145337>.

[4] Q. Ouyang, C. Yao, H. Chen, L. Song, T. Zhang, D. Chen, L. Yang, M. Chen, H.J. Chen, Z. Peng, X. Xie, Machine learning-coupled tactile recognition with high spatiotemporal resolution based on cross-striped nanocarbon piezoresistive sensor array, Biosens. Bioelectron. 246 (2024) 115873. <https://doi.org/10.1016/j.bios.2023.115873>.

[5] N.T. Beigh, F.T. Beigh, D. Mallick, Machine learning assisted hybrid transduction nanocomposite based flexible pressure sensor matrix for human gait analysis, Nano Energy 116 (2023). <https://doi.org/10.1016/j.nanoen.2023.108824>.

[6] J. Chen, X. Xia, X. Yan, W. Wang, X. Yang, J. Pang, R. Qiu, S. Wu, Machine Learning-Enhanced Biomass Pressure Sensor with Embedded Wrinkle Structures Created by Surface Buckling, ACS Appl Mater Interfaces 15(39) (2023) 46440-46448. <https://doi.org/10.1021/acsami.3c06809>.

[7] H. Yang, J. Li, X. Xiao, J. Wang, Y. Li, K. Li, Z. Li, H. Yang, Q. Wang, J. Yang, J.S. Ho, P.L. Yeh, K. Mouthaan, X. Wang, S. Shah, P.Y. Chen, Topographic design in wearable MXene sensors with in-sensor machine learning for full-body avatar reconstruction, Nat Commun 13(1) (2022) 5311. <https://doi.org/10.1038/s41467-022-33021-5>.

[8] A. Moin, A. Zhou, A. Rahimi, A. Menon, S. Benatti, G. Alexandrov, S. Tamakloe, J. Ting, N. Yamamoto, Y. Khan, F. Burghardt, L. Benini, A.C. Arias, J.M. Rabaey, A wearable biosensing system with in-sensor adaptive machine learning for hand gesture recognition, Nature Electronics 4(1) (2020) 54-63. <https://doi.org/10.1038/s41928-020-00510-8>.

[9] R. Zhang, X. Chen, Z. Wan, M. Yin, L. Ma, Z. Yang, X. Xiao, High-Performance, flexible moist-electric generator for self-powered wearable wireless sensing, Chemical Engineering Journal 502 (2024). <https://doi.org/10.1016/j.cej.2024.157695>.

[10] H. Xu, W. Zheng, Y. Zhang, D. Zhao, L. Wang, Y. Zhao, W. Wang, Y. Yuan, J. Zhang, Z. Huo, Y. Wang, N. Zhao, Y. Qin, K. Liu, R. Xi, G. Chen, H. Zhang, C. Tang, J. Yan, Q. Ge, H. Cheng, Y. Lu, L. Gao, A fully integrated, standalone stretchable device platform with in-sensor adaptive machine learning for rehabilitation, Nat Commun 14(1) (2023) 7769. <https://doi.org/10.1038/s41467-023-43664-7>.

[11] M. Wang, T. Wang, Y. Luo, K. He, L. Pan, Z. Li, Z. Cui, Z. Liu, J. Tu, X. Chen, Fusing Stretchable Sensing Technology with Machine Learning for Human–Machine Interfaces, Advanced Functional Materials 31(39) (2021). <https://doi.org/10.1002/adfm.202008807>.

[12] S.K.H. Hee Seung Wang, Jae Hyun Han, Young Hoon Jung, Hyun Kyu Jeong Tae Hong Im, Chang Kyu Jeong, Bo-Yeon Lee, Gwangsu Kim, Chang D. Yoo, Keon Jae Lee, Biomimetic and flexible piezoelectric mobile acoustic sensors with multiresonant ultrathin structures for machine learning biometrics, Science advances 7 (2021).
